# Factors Influencing the Adoption of Smart Health Technologies for People With Dementia and Their Informal Caregivers: Scoping Review and Design Framework

**DOI:** 10.2196/12192

**Published:** 2019-04-30

**Authors:** Estefanía Guisado-Fernández, Guido Giunti, Laura M Mackey, Catherine Blake, Brian Michael Caulfield

**Affiliations:** 1 University College Dublin School of Public Health, Physiotherapy and Sports Science Dublin Ireland; 2 Insight Centre for Data Analytics University College Dublin Dublin Ireland; 3 University of Oulu Oulu Finland

**Keywords:** dementia, informal caregiver, smart health technologies, user-centered design, technology adoption

## Abstract

**Background:**

Smart Health technologies (s-Health technologies) are being developed to support people with dementia (PwD) and their informal caregivers at home, to improve care and reduce the levels of burden and stress they experience. However, although s-Health technologies have the potential to facilitate this, the factors influencing a successful implementation in this population are still unknown.

**Objective:**

The aim of this study was to review existing literature to explore the factors influencing PwD and their informal caregivers’ adoption of s-Health technologies for home care.

**Methods:**

Following the Arksey and O’Malley methodology, this study is a scoping review providing a narrative description of the scientific literature on factors influencing s-Health technology adoption for PwD and their informal caregivers. A search was conducted using PubMed, the Cochrane library, the IEEE library, and Scopus. Publications screening was conducted by 2 researchers based on inclusion criteria, and full-text analysis was then conducted by 1 researcher. The included articles were thematically analyzed by 2 researchers to gain an insight into factors influencing adoption that PwD and their informal caregivers have to encounter when using s-Health technologies. Relevant information was identified and coded. Codes were later discussed between the researchers for developing and modifying them and for achieving a consensus, and the researchers organized the codes into broader themes.

**Results:**

Emerging themes were built in a way that said something specific and meaningful about the research question, creating a list of factors influencing the adoption of s-Health technologies for PwD and their informal caregivers, including attitudinal aspects, ethical issues, technology-related challenges, condition-related challenges, and identified gaps. A design framework was created as a guide for future research and innovation in the area of s-Health technologies for PwD and their informal caregivers: *DemDesCon for s-Health Technologies*. DemDesCon for s-Health Technologies addresses 4 domains to consider for the design and development of s-Health technologies for this population: cognitive decline domain, physical decline domain, social domain, and development domain.

**Conclusions:**

Although s-Health technologies have been used in health care scenarios, more work is needed for them to fully achieve their potential for use in dementia care. Researchers, businesses, and public governments need to collaborate to design and implement effective technology solutions for PwD and their informal caregivers, but the lack of clear design guidelines seems to be slowing the process. We believe that the DemDesCon framework will provide them with the guidance and assistance needed for creating meaningful devices for PwD home care and informal caregivers, filling a much-needed space in the present knowledge gap.

## Introduction

### Background

Dementia is a neurodegenerative chronic condition characterized by a progressive decline in a person’s memory, thinking, learning skills, and ability to perform activities of daily life (ADLs). Currently, dementia affects 47 million people worldwide, and these numbers are expected to increase to 75 million in 2030 and 132 million by 2050 [1]. As a result, the World Health Organization has declared it a public health priority and launched a public health plan in 2017 [2]. A diagnosis of dementia also has a significant impact on family members of people with dementia (PwD), who often bear the responsibility of caring for them as their health deteriorates [3]. Individuals who provide unpaid and continuous assistance and have not been formally trained, such as spouses, children, or other family members, are referred to as *informal caregivers*, in contrast to *formal caregivers*, who offer paid professional services [3]. Furthermore, it is often the case that informal caregivers provide care to PwD in circumstances where formal health care does not reach because of health care systems infrastructure, socioeconomic status, or cultural preferences, among others [4].

Smart Health technologies (s-Health technologies) [5] *are the result of the natural synergy between m-Health and smart cities, from the Information and Communication Technologies (ICT) perspective, as well as that of individuals and society*. Nowadays, a wide variety of s-Health technologies are being developed to help the elderly, chronic patients, and their informal caregivers at home, showing promising results [6,7]. The use of s-Health technologies for dementia includes assisted living technology, ambient assisted living technologies, and smart homes. Cahill et al proposed [8] that s-Health technologies fall into 4 main categories, namely, (1) those used to promote safety, (2) those that foster communication and address memory loss problems, (3) those that provide multisensory stimulation, and (4) those that act as memory enhancers. The scientific literature points out that s-Health technologies may have a role in supporting informal caregivers of PwD for situations often associated with informal caregiving, such as symptoms of depression, stress and anxiety, or caring burden [9,10]. Recent research on the topic [11], however, has found that many of these systems fail to be effective in real-life cases because of their low acceptance and adoption, often relating this to usability issues.

Technology can facilitate the delivery of care, but there are certain factors that can diminish its effectiveness. Obstacles to adoption are many and can range from design choices to complex scenarios like potential ethical issues such as data ownership or privacy concerns derived from their use [12]. In terms of usability, challenges increase for dementia as we must also consider the cognitive and behavioral issues [13]. For PwD, even once familiar devices, such as washing machines, microwaves, kettles, or telephones, can be problematic, as the appearance and design of these have changed so much that they do not resemble the ones they had grown accustomed to [14].

### Gaps in the Knowledge

Current trends in health information technologies suggest that solutions should be designed not only to be effective, acceptable, and nonharmful but also to be pleasant and engaging [15,16]. The use of user-centered design (UCD) principles generates systems that are easy to learn, have higher user acceptance and satisfaction, and lower user errors [17-19]. Design for PwD should consider dementia-related symptoms [13]. Furthermore, informal caregivers of PwD provide substantial care at home, at times with little assistance from paid professionals [3]. Informal caregivers of PwD spend large amounts of time caring for PwD and are very acquainted with the problems they face [20]. However, they are seldom included in the design process for s-Health technologies, which could prove beneficial.

To the best of our knowledge, there are no guidelines specifically created for designing s-Health technologies for PwD and their informal caregivers. There are sets of design recommendations such as the one created by Astell et al for *motor-based technologies for people with cognitive impairment* [21], Boman et al’s work on using ICTs for persons with cognitive impairment [22], or Matthews et al’s [23] summary of adoption factors for caregivers of dementia. However, these recommendations do not expand on the process of design, develop, and most of all, implementation of s-Health technologies [21].

The objective of this study was to examine the factors influencing PwD and their informal caregivers’ adoption of s-Health technologies for home care and provide some recommendations for their design.

## Methods

### Study Design

Scoping review methodology aims to map the key concepts underpinning a research area, especially where an area has not been reviewed comprehensively before [24-26]. The Arksey and O’Malley methodology [24] was followed to produce a scoping review that provides a narrative description of the scientific literature on factors influencing s-Health technology adoption for PwD and their informal caregivers. A qualitative thematic analysis [27] was conducted on the results of the scoping review to generate a list of design recommendations that aim to help future s-Health technologies for PwD and their informal caregivers’ researchers and designers.

### Identifying the Research Question

The aim of this study was to review existing literature to explore the factors influencing PwD and their informal caregivers’ adoption of s-Health technologies for home care.

### Identifying Relevant Studies

We conducted a search on available literature on s-Health technologies for PwD and their informal caregivers following the selection criteria (see below). The search was conducted in the following databases: PubMed, Cochrane library, IEEE library, and Scopus. Initially, titles and abstracts of all publications retrieved from the initial search were screened by 2 researchers (EGF and LMM), and a full-text analysis of potentially suitable publications was then conducted by 1 researcher (EGF).

Inclusion criteria were as follows:

1. Publications in English language.
2. Literature that dealt with PwD and their informal caregivers.
3. s-Health technologies interventions that were designed, implemented, or evaluated for PwD and their informal caregivers in outpatient scenarios.
4. Publications that included primary or secondary outcome evaluations on usability and user experience, adoption barriers and enhancers, design, participant’s level of satisfaction with the technology, and technology friendliness.

Exclusion criteria were as follows:

1. Studies that took place in nursing homes or care facilities.
2. Young-onset dementia studies.

The keywords and search terms used were organized into 3 main groups for clarity purposes: (1) dementia dementia-related keywords, (2) informal caregiver–related keywords, and (3) s-Health technologies–related keywords. A complete list of all keywords and the search string can be found in Multimedia Appendix 1.

## Results

### Study Selection

In total, 2 researchers (EGF and LMM) completed the study selection process; disagreements were resolved by involving a third researcher (CB). The selection and analysis process was managed with EndNote X8 software for Mac (Clarivate Analytics, Philadelphia).

**Figure 1:**
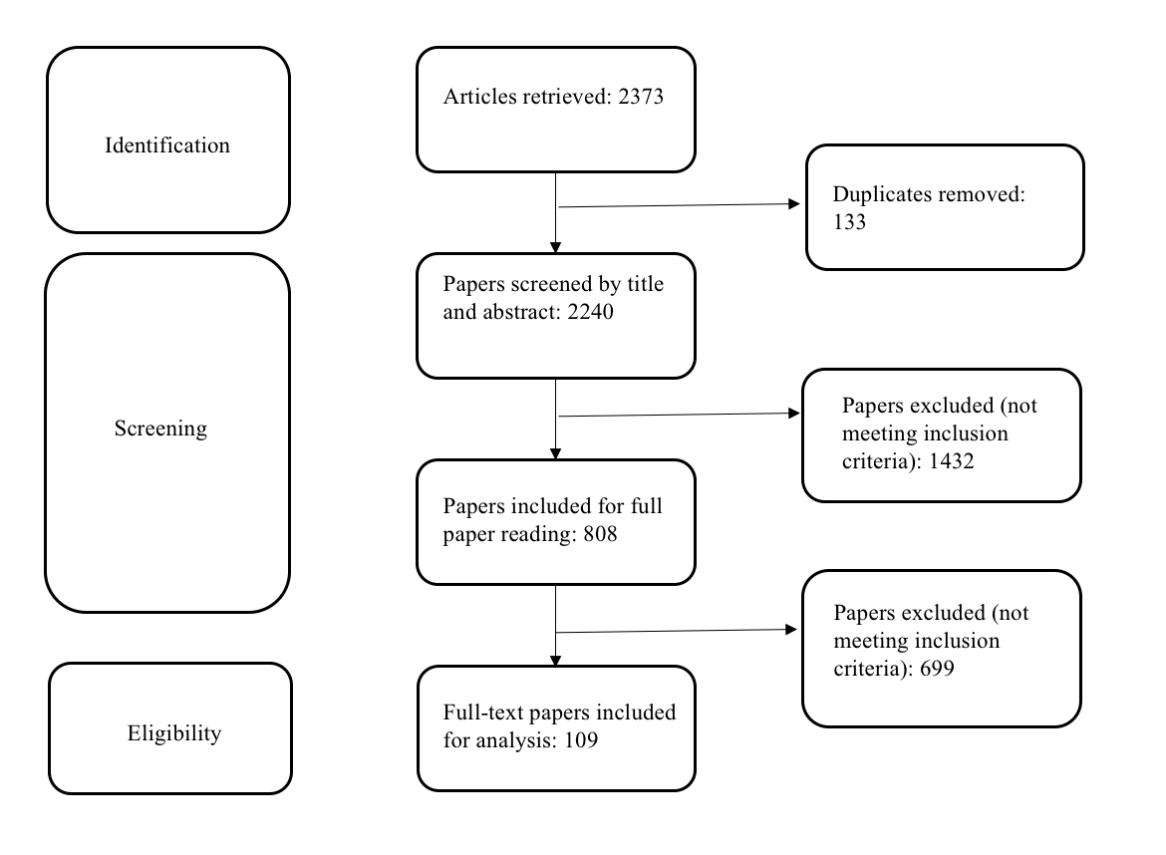
Study selection workflow.

Our search strategy retrieved 2373 publications from the selected sources. After removing duplicates, publications were screened by title and then by abstract, which identified 808 papers to read by full text. A total of 109 publications met our inclusion criteria and were included for analysis. The study workflow selection can be seen in Figure 1.

### Charting the Data

**Figure 2:**
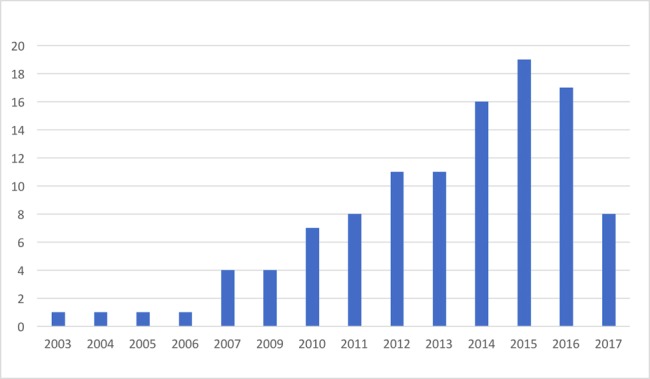
Numbers of papers identified per year.

Data extraction forms were created to obtain and organize information from the included studies, such as author(s), year and journal of publication, title, type of publication, study design and aim, target population, intervention, the type of technology included, outcomes, and potential technology adoption challenges described. A summary of the included publications can be seen in Table 1 and Figure 2.

**Table 1:** Publications summary table.

Characteristics		n
**Type of publication**		
	Design studies	56
	Book chapter	1
	Review studies	8
	Randomized controlled trials	6
	Nonrandomized controlled trials	1
	Qualitative studies	10
	Case studies	3
	Pilot studies	19
	Longitudinal studies	2
	Exploratory studies	2
	Cross-sectional studies	1
**Type of technology included**		
	Communication and Information Technologies (ie, electronic health, mobile health, telehealth, telecare, and home monitoring)	47
	Assisted living technologies (ie, pervasive assistive technology, ambient assisted living technologies, and smart interactive artifacts)	56
	Health smart home	7
	Wearables and tracking devices	9

**Table 2:** Factors influencing the adoption of s-Health technologies for people with dementia and their informal caregivers.

Theme 1: Attitudinal aspects	Theme 2: Ethical issues	Theme 3: Technology-related challenges	Theme 4: Condition-related challenges	Theme 5: Gaps
Positive attitudes toward technology: improved quality of life; self-efficacy facilitator; and support tool. Negative attitudes toward technology: lack of technology acceptance; mismatched expectations and needs; and technology burden	Privacy concerns; autonomy concerns; and data ownership concerns	Design; digital literacy; and perceived usefulness	Cognitive decline; aging and physical capabilities; and condition acceptance	Market needs; research; and good practice guidelines

### Collating, Summarizing, and Reporting the Results

Only qualitative and narrative data that could be obtained from the studies were included. The qualitative analysis was performed using NVivo software version 12 for Mac (QSR International, Melbourne, Australia). Data analysis was conducted independently by the 2 main researchers (EGF and LMM). Through the iterative process, relevant information was identified and coded. Codes were later discussed between the researchers for developing and modifying them, achieving a consensus. Researchers organized the codes into broader themes that said something specific and meaningful about the research question. Finally, a list of design considerations was created based on our findings, as a guide for future research and innovation in the area of s-Health technologies for PwD and their informal caregivers.

### Emerging Themes

In this section, we provide a detailed description of the emerging themes and factors influencing the adoption of s-Health technologies for PwD and their informal caregivers that rose from our qualitative analysis of the included results. An overview is presented in Table 2.

### Theme 1. Attitudinal Aspect

A user’s attitude toward technology is crucial for its adoption. Having a positive attitude toward technology and having previous positive experiences are known to improve adoption [28,29]. Negative attitudes can act as barriers, resulting in nonadoption [8].

#### Positive Attitudes Toward Technology

##### Improved Quality of Life

Some informal caregivers see s-Health technologies as means to improve QoL through better care management, reducing their levels of anxiety, burden, and frustration, and also impacting positively on the QoL of PwD [30]. s-Health technologies are also seen to improve the ability to cope with challenging situations that arise from living with the condition [31]. There seems to be some empowerment happening as the costs and time saved by using the technology allow informal caregivers to gain more freedom [12,32,33]. For example, as shown in the study by Mitseva et al [32], being able to avoid the hassle of going over to the care centers or visiting the PwD’s home was afforded by technology.

##### Self-Efficacy Facilitator

In some papers, using technologies is considered to promote PwD independent living, as it reassures both PwD and informal caregivers. s-Health technologies were able to provide entertainment and engage PwD [34,35], making it easier for PwD to stay out of hospice care while increasing informal caregivers’ peace of mind [36-38].

##### Support Tool

Communication between PwD and informal caregivers can be greatly improved through s-Health technologies [8,30,34]. These technologies can enhance the user’s social network and the support they provide [39]. Gradually introducing technologies to PwD’s lives increased the chances for their adoption for ADLs, helping in their use for later stages of the disease. As mentioned in the study by Patterson et al [40], technologies even become invisible for the PwD maximizing its integration. A sudden introduction is recognized as a barrier, as it can make PwD reject technology [41,42].

#### Negative Attitudes Toward Technology

##### Lack of Technology Acceptance

The overall feeling from the literature seems to be that the elderly are reluctant users who do not engage with newer technology [43]. This may be so depending on the stage of the condition. Some studies presented the view that PwD do not see themselves as ideal users, either because they do not feel the technology is suitable for them or because they think that *they are not that bad* [39,44].

##### Mismatched Expectations and Needs

In the literature, it is common to find that PwD and informal caregivers have unrealistic expectations regarding what s-Health technologies can accomplish for them [31,45,46]. This is one of the most common perceptions as technologies are not considered sufficiently well suited to their needs [45] or they expect more than what technology can currently offer [31,46].

##### Technology Burden

Many negative feelings may arise from technology use, such as frustration, confusion, discomfort, embarrassment, or anxiety [14,37,47], which may have impacts on technology adoption. Some studies found that using reminder systems can be burdensome to informal caregivers, who continuously had to remind PwD to use the device [8,48,49]. Technology use also carries routine disturbance [43,50], fear of becoming dependent on technology, and fear of the informal caregivers being replaced by machines [51].

### Theme 2. Ethical Issues

The use of s-Health technologies is not without ethical concerns as issues of autonomy, beneficence, and justice, among other moral issues, can be presented. Questions such as PwD’s ability to provide truly informed consent, how is their privacy protected, or how confidential the information given is are present throughout the literature [52].

#### Privacy Concerns

Lack of privacy is described as a major issue for both PwD and informal caregivers, which is seen as a potential risk that could stigmatize them and take away their dignity [53,54]. Informal caregivers were usually putting PwD safety needs first over any other concern, believing that remote tracking could reassure them as caregivers [55], but they feared that it would be obtrusive to personal lives and wanted the option to turn it off [56,57].

#### Autonomy Concerns

Similar to the theme above, this concern is related to the fear that constant monitoring of PwD is restricting their freedom [53,54]. Preserving some semblance of autonomy was important as the loss of personal freedom can lead to the infantilization of PwD [58].

#### Data Ownership Concerns

Through the use of s-Health technologies, new and vast amounts of data are generated; who does it belong to seemed to be a frequent question. Preserving the confidentiality of sensitive data and preventing exposing it in any personally identifiable way was very important [59]. To avoid this, best practices in dementia research recommend the involvement of PwD [33].

### Theme 3. Technology-Related Challenges

Technology is becoming part of PwD and informal caregivers’ daily lives, but many devices require a number of different tasks to be performed for them to function properly. There are certain aspects that need to be addressed for the PwD and their informal caregivers to feel that s-Health technologies were not *dropped* into their lives with little to no information or guidance on how to use it [36].

#### Design

The design process plays an important role in its use, acting as a barrier or a facilitator. In terms of the devices’ external aspect, overly bulky or too conspicuous gadgets can result in the technology being abandoned [8,60]. Smallness and discreteness for home-installed and body-worn devices were considered less stigmatizing in the literature [22,59,61,62]. Furthermore, it seems that PwD respond better to devices that have a familiar aspect [8,59], for example, televisions (TVs) with adapted interfaces to mimic older sets [41]. In regard to user interface design, the most frequent recommendation is that it should be user-friendly, simplified, and easy and clear to use [11,44,61,63-66]. This includes considerations such as appropriate colors, text font and sizes, and background styles and sounds, adapted to fit PwD’s hearing and vision common problems [67,68]. Being able to tailor the technology to match PwD cognitive and health status is important as the disease progresses [36,69,70]. In addition, s-Health technologies that allow tailored content, such as pictures or components to make it *fun* to use, are considered less stigmatizing and hence more likely to be embraced [71]. Involving PwD and informal caregivers in the design process enhanced usability and technology acceptance [59,72].

#### Digital Literacy

The literature points out that PwD and informal caregivers are often unaware about what technology can do for them and what it can do to help in their daily activities [38,43,73]. The lack of information can act as a barrier, and proper supply can be a facilitator. Digital literacy [40,44] is so important that not being educated about it could increase the need for additional time and efforts to adopt a new device [40,74,75]. The lack of digital literacy was somehow mitigated when caregivers and PwD used technology together [76].

#### Perceived Usefulness

It is a recognized issue that target users of technology need to see it as valuable to adopt it [59,73,77]. This was true for both PwD and informal caregivers, who want to know this before even considering purchasing them [23,78]. These were some of the main issues associated with drop out from s-Health technologies studies [79]. Usefulness and cost are closely associated, as users tend to be surer about purchasing an s-Health technologies device when the price is low, as throwing them away will be less painful [73].

### Theme 4. Condition-Related Challenges

There are a series of issues that PwD face as a result of living with the condition and the natural age-related changes. This gradual deterioration affects the performance of specific functional tasks as well as cognitive deficits that impair learning new systems and interfaces, impacting the interaction with new technologies [40].

#### Cognitive Decline

The nature of dementia can greatly result in active rejection of technology. There were several studies that were related in part to memory decline and aging-related problems such as hearing or vision [40]. PwD were more suspicious of new things [13]. In addition, condition denial is a factor for PwD as they generally do not wish to be reminded of their condition [30]. In the late stages of the condition, PwD have greater difficulties making decisions for themselves. This creates conflict for informal caregivers, who have to balance their own personal needs (eg, peace of mind) and the potential infringement of PwD’s autonomy and independence [36,59]. There is literature that supports the involvement of PwD in decision making whether or not they have been legally or clinically deemed unfit [28]. Deciding early to what degree of decline PwD can continue participating in an intervention was a highlighted matter [43].

#### Aging and Physical Capabilities

Physical changes associated with aging, such as sight and hearing loss, health issues, or aging tremor, can impact the adoption of s-Health technologies. Using certain touch screens, keyboards, fonts, button sizes, colors, and design can be troublesome [8,40,79,80]. Considering issues with fine motor skills, flexible and intuitive technologies that require minimal physical effort [22] and minimize the need for interactions [76] are more appropriate. Optimizing the number of functions and features that can be integrated into each system makes it easier and simpler to use [71].

#### Condition Acceptance

As mentioned earlier, the lack of awareness and the disease denial attitude that this population usually has at the early stages imply a lack of recognition of their disabilities and needs, and therefore a rejection of any kind of help, including s-Health technologies [43]. However, in the case of the informal caregivers, they report that home care technology provides the PwD with a greater understanding and perception of the disease, enhancing the diagnosis acceptance [81].

### Theme 5. Gaps

There is a common concern in the literature that there are many gaps in terms of the market availability of technological developments for PwD and informal caregivers, and lack of practical guidelines for the design and implementation of technologies [14].

#### Market Needs

In some aspects, market size may determine how much research is conducted. PwD and their informal caregivers are a relatively small percentage size compared with other health condition populations, and perhaps this explains the limited attention that the design and evaluation of technologies has received for this population [82].

#### Research

In general, technologies are designed by cognitively intact people, such as system developers, researchers, and their colleagues [82]. There is a noticeable lack of involvement of PwD and informal caregivers in research despite the fact that academic and industrial sectors claim how important this would be to avoid s-Health technology nonadoption or abandonment [33,63,72]. More research is needed to determine the appropriate level of interaction between the PwD and informal caregivers with the different technologies depending on the disease stage [59], and to determine whether successful outcomes are disease-related, age-related, or both [32].

#### Good Practice Guidelines

It is evident from the literature that there is a great need for guidelines on how to design and develop technological solutions for this population [31,83,84].

### DemDesCon for s-Health Technologies: Dementia Design Considerations for Smart Health Technologies

**Figure 3:**
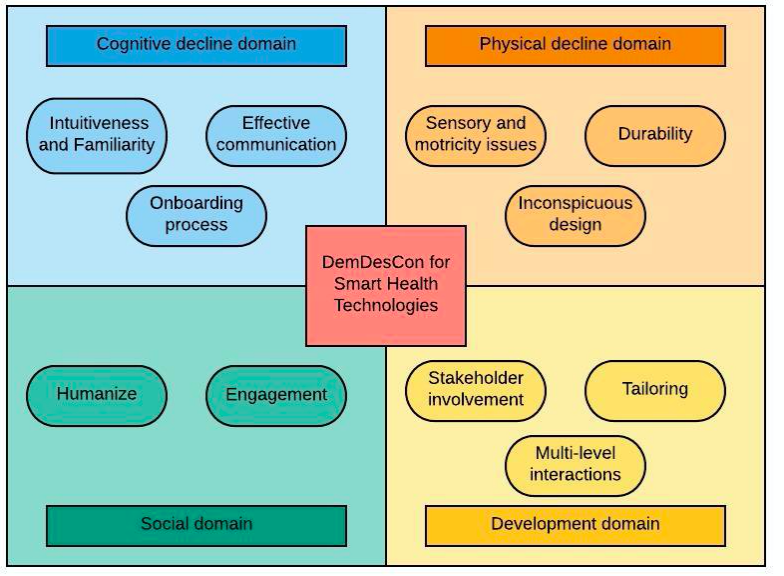
Dementia Design Considerations for Smart Health Technologies (DemDesCon for s-Health technologies).

The emerging themes obtained during our scoping review allowed the extraction of valuable insight that was grouped to create a series of design considerations for s-Health technologies for PwD and informal caregivers. We have called these Dementia Design Considerations for Smart Health Technologies or *DemDesCon for s-Health technologies*. Following the works of Astell et al [21], we have presented these design considerations as different domains to be taken into account. Each design consideration is detailed below. An overview of DemDesCon for s-Health technologies is presented in Figure 3.

Designers of s-Health technologies are encouraged to consider these different domains in their approach to the design process of solutions for PwD and informal caregivers and to reflect on the ramifications of their designs.

### Cognitive Decline Domain

PwD undergo a series of cognitive decline issues that affect how they can relate to new technologies. The following sections reflect on design considerations regarding their cognitive capabilities.

#### Intuitiveness and Familiarity

PwD have a hard time acquiring new knowledge or developing new learning skills; hence, taking advantage of their preserved skills is considered that can facilitate this acquisition process [34,59]. In this case, the old saying of *less is more* seems to work better here. s-Health technologies interfaces have to be easy to use, clear, not complex, and as simple as possible [8,11,44,64,85,86]. s-Health technologies should encourage interaction [34] and have a uniform composition, paying attention to font type and size, colors, and shape of buttons [44,65,87]. As it is with hardware, the interface should emphasize *recognition rather than recall*, thus being easier, quicker, and better appreciated by the users [65].

One approach that is recommended for s-Health technologies is to adopt a familiar look that mimics older devices. This is because learning new things is not a preserved skill for the PwD [13,88]. Using devices that mimic technology that PwD already use, such as old-fashioned TV screens, radios, or phones, have proved to have increase adoption [14]. s-Health technologies devices should aim to be comforting and nonthreatening to avoid them being rejected [89,90].

#### Effective Communication

Use of plain and common language is a must, to avoid PwD confusion [44] and to facilitate understanding and interaction. It is common in human nature that when we cannot understand something, we tend to abandon it or are reluctant to engage with it. The content provided should be tailored as well [44], providing well-distributed information and divided into different modules depending on each individual’s needs [87,88].

Setting simple and achievable goals or tasks with clear and appropriate instructions increases the chances that PwD’s cognitive impairment will not impede to carry them out [23]. To engage and retain PwD’s attention, a certain negotiation and interpretation needs to be done to present tasks in a way that seems attractive to the PwD [34].

#### Onboarding Process

Introducing new technologies in the life of cognitively impaired people such as PwD should be done with careful consideration [91]. In early stages of the cognitive decline, cognitively impaired people are still able to provide consent about whether to use a piece of technology or not, and in later stages, it is recommended that their former wishes regarding having technology used for their care are taken into account [28].

Enough time should be given to PwD and informal caregivers to get used to using them, providing time to learn at their own pace and suiting preserved cognitive skills. Providers should provide guidance on how to use s-Health technologies [39] and allow sufficient time to practice [92]. In the event of system failures, home assistance should be provided, avoiding further disruptions of PwD or informal caregiver’s life.

### Physical Decline Domain

The gradual and progressive physical deterioration that PwD go through requires for potential s-Health technologies to take special notice to some particular condition-related aspects. Below are design considerations relevant to the physical decline of PwD.

#### Sensory and Motricity Issues

As PwD fine motor skills decline, using appliances or devices such as a computer mouse or TV remote control, can represent a struggle 34]. Adapting visual and audio signals to compensate these issues is recommended [23], for example, providing larger screens [34] or easily adjustable volume settings [8].

#### Durability

As PwD’s condition deteriorates, they are more prone to destructive behaviors such as lashing out [90]. This is in part because of their lost motor skills and lack of cognitive processing as well as other disease-related problems such as their lost learning capabilities [59].

Taking these issues into account, the literature recommends that s-Health technologies for PwD be of robust materials or, in the event of wearable devices, not easy to be removed from clothes, belt, or body, to withstand these outbursts or potential neglect [38].

#### Inconspicuous Design

In line with the above mentioned information, portable and ergonomic devices are a good approach for the design of s-Health technologies for PwD [61,78]. It is also preferable that these devices be lightweight and comfortable to wear or carry and have reduced dimensions and discrete designs, as PwD are likely to have to carry them to frequent places such as a health care provider’s office or family members’ houses or even during vacations [23,91]. These recommendations facilitate and increase wearability and adoption [62,88]. Inconspicuous designs reduce feelings of stigmatization [49], unlike body-worn devices that are very noticeable, such as pendant alarms or Global Positioning Systems trackers.

The different locations and scenarios in which these s-Health technologies can be used present a challenge [71] as these devices may have charging requirements that can be bothersome, causing abandonment [8].

### Social Domain

It is common for people and health care providers to, sometimes without noticing, infantilize PwD, thus taking their autonomy away from them. The following design considerations attempt to emphasize ways in which s-Health technologies can encourage PwD to stay active and offer them positive reinforcement.

#### Humanize

s-Health technologies in this field should try to support PwD, not seeing them as just mere objects or former humans that have lost their memory and abilities [93]. Technology should promote social interaction and avoid isolation [41]. Loneliness and sadness are very common in PwD and their informal caregivers; because of the disease progression, their social network keeps reducing, rendering them even more isolated.

s-Health technologies should aim to promote autonomy, making the PwD more independent and self-reliable in their ADLs [94]. A more independent PwD will give more free time to informal caregivers themselves, allowing them to engage in leisure and social activities as well as keeping physically and socially active.

#### Engagement

The content of the activity or intervention must be suited to meet PwD and informal caregivers’ personal interest [76], for example, using audio-visual media such as photos, videos, or music that are appealing to them. In this case, the content should be customized for the PwD’s own interests, hobbies, or preferences.

### Development Domain

s-Health technologies are becoming more pervasive, but there is much room left for improvement in regard to creating solutions that are useful and meaningful for the intended audiences. The following design considerations aim to call for attention from researchers, developers, and designers as to what seems to be missing in the field of s-Health technologies for PwD.

#### Stakeholder Involvement

It is advisable to involve PwD and their informal caregivers in all phases of s-Health technologies design, as their input will enhance the suitability and acceptability of the solution as well as empower them [59,70,88]. Stakeholder involvement also helps to establish closer and more trustful relationships, to understand the needs and values of all stakeholders, and overall, adds value to the design [95].

#### Tailoring

Tailoring is a process for creating individualized communications by gathering and assessing personal data related to a given health outcome in order to determine the most appropriate strategy to meet patient’s unique needs [96,97]. Compared to generic information, tailored information is more likely to be read, remembered and viewed as personally relevant [98]. Therefore, s-Health technologies have to reach the users in a way that is meaningful to them, being able to adapt to the different stages of the disease and the symptoms fluctuation, as well as to their physical and mobility decline, offering different solutions depending on the needs and assistance required, moving from an active user to a passive user when necessitated [74].

This tailoring is desired to be automatic, that is, the device works by itself with little or no direct human control, deciding the type and level of help required without the informal caregiver or the PwD intervention [39,74,99]. It has to allow to set up tasks and also make them adaptable to changes to patient’s situation fluctuations and to not interfere with their daily routines [50,100]. This means that technology has to be as much flexible as possible and cannot be outdated as the disease progresses [50].

#### Multilevel Interactions

s-Health technologies for PwD should allow different levels of intervention, differentiating between health care professionals, other peers, family, and relatives [101]. The user has to be able, if desired, to personalize the support, information requested and shared, levels of assistance, and communication with different members of the support circle [81].

## Discussion

### Principal Findings

This scoping review is the first study of its kind to explore factors influencing the adoption of s-Health technologies for PwD and informal caregivers. A total of 109 papers were reviewed and thematically analyzed, providing insight into factors influencing s-Health technologies adoption when using these types of technologies for the home care environment. Emerging themes were divided and classified for better understanding. Furthermore, the insights that this review provides were used to produce a series of design considerations for future work in s-Health technologies for dementia home care. No other study has provided a similar list of design considerations for use in s-Health technologies for dementia before.

### Comparison With Previous Work

A thorough review of the scientific literature highlights how previous works where technology has been used for dementia home care leave room for confusion with an ample array of terms and terminologies employed. The lack of a uniform nomenclature or taxonomy becomes apparent as a variety of concepts are frequently used together, even within the same publications, making it quite difficult to distinguish which type of technology is being referenced [102,103].

s-Health technologies for PwD and informal caregivers seem to be created nowadays with either the PwD [77] or the informal caregivers [44,81,104] in mind. It is less common in the cases where the services are being designed for both of them and integrated under the same tool [105,106]. This is in conflict with recommendations for designing technology in this population, as one of the essential points is the comprehensive creation and a participatory design [14,95]. Despite these issues and facing great difficulties, PwD and informal caregivers appear to be embracing dementia home care technologies slowly yet increasingly to facilitate and to assist them with their ADLs.

Another interesting finding in our scoping review is the significant emphasis that studies placed on feasibility, reliability, usability and user experience, and user engagement, regardless of overall goals of behavioral change. It would seem that the latter was not often accounted for or corroborated in the results. Furthermore, the methodology used in the studies varied even for assessing similar variables, making it difficult to extrapolate conclusions [107].

Working up from the gaps and themes that we discovered in our study, we turn our attention to the actual s-Health technologies design for PwD. Current trends in s-Health technologies design claim that UCD processes increase their adoption and use by the intended users [17]. In UCD, the needs and perspectives of users are placed in the highest of priorities and the product is designed to accommodate them [19]. Ideally, this should be an iterative design process, where the final users contribute with their knowledge and experience to develop a product that can be adapted to meet their own needs in a user-friendly manner. By following these design principles, systems that are easier to learn, have higher user acceptance and satisfaction, and have lower user errors are generated [17-19].

The design of s-Health technologies for PwD unfortunately does not follow the above approaches. In many cases, PwD and informal caregivers are not involved during the design process, so the value of their experiences and expertise is lost. It is more likely that they are involved in a user evaluation exercise that takes place after the design process has already reached an advanced stage. Thus, intended users have to make a great effort to understand and become familiar with the particularities of each device. In many cases, this is not successful, resulting in the device being abandoned for not being found useful or fit for its purpose [31]. There is a necessity to better understand the needs and perceptions of PwD and informal caregivers regarding technology, and to use this knowledge to address the deficits outlined above. Therefore, we believe that our work will provide light in those gray and diffuse areas where there are no guidelines at the moment.

As it stands, DemDesCon for s-Health technologies is aligned with current design models for technologies that advocate the need to consider condition-specific factors [108,109]. In addition, some frameworks also suggest involving stakeholders to ensure that the designed technology is more meaningful to end users [110]. We have included 28 publications [111-139] in Multimedia Appendix 1.

### Limitations

One of the main limitations of this study relies on its research methodology, as scoping reviews do not explore the totality of all available studies; rather, it provides a descriptive view of the area of study. In addition, the scoping reviews do not seek for quality and weight of evidence or quality of the methodology of the primary research publications. It also has to be considered that the amount of information collected can lead into difficulties for the width and depth of the information to cover.

Furthermore, no quantitative or statistical analysis was performed on the included papers, but this is in line with the scoping review methodology. It is possible that the selection criteria may have left out studies that would be relevant to this research’s goal, such as non-English publications that could hold relevant studies in other languages. Focusing on community-living PwD and their informal caregivers may have neglected other suitable studies conducted in caring homes or in other types of people with other chronic diseases that could also benefit from these types of interventions. In addition, young-onset dementia interventions have not been taken into account, and this could be a bias as we have mentioned in our paper; an early introduction is a key factor for technologies adoption.

### Conclusions

Although s-Health technologies have been used in health care scenarios, more work is needed for them to fully achieve their potential for use in dementia care. As was present in the revised literature, s-Health technologies are seen by some as a complementary support tool that could improve the quality of life (QoL) of PwD and informal caregivers, who are willing to use these technologies if the conditions are right.

Our study found that the way of matching the appropriate technology to each individual, and at the right time, is not clear yet and more difficult than what it may seem. Researchers and companies are working toward developing valuable technologies for PwD and informal caregivers, but the lack of clear design guidelines seems to be slowing the process. This study offers a series of design recommendations under the shape of a framework: *DemDesCon for s-Health technologies*. We believe DemDesCon can provide guidance and assistance for creating meaningful devices for home care for PwD and informal caregivers, filling a much-needed space in the gap of knowledge. Nevertheless, more research needs to be conducted with longitudinal studies to appreciate how s-Health technologies work in the users’ environment and how they interact with them.

## Appendix

Multimedia Appendix 1 Annexes.

## Abbreviations

ADL: activity of daily life
s-Health technologies: Smart health technologies
PwD: People with Dementia
QoL: quality of life
TV: television
UCD: user-centered design

## References

[1] (2012). Dementia: A Public Health Priority.

[2] (2017). Global Action Plan on the Public Health Response to Dementia 2017-2025.

[3] Donelan K, Hill CA, Hoffman C, Scoles K, Feldman PH, Levine C, Gould D (2002). Challenged to care: informal caregivers in a changing health system. Health Aff (Millwood).

[4] (2016). Families Caring For An Aging America.

[5] Solanas A, Patsakis C, Conti M, Vlachos I, Ramos V, Falcone F, Postolache O, Perez-martinez P, Pietro R, Perrea D, Martinez-Balleste A (2014). Smart health: a context-aware health paradigm within smart cities. IEEE Commun Mag.

[6] Chouvarda IG, Goulis DG, Lambrinoudaki I, Maglaveras N (2015). Connected health and integrated care: toward new models for chronic disease management. Maturitas.

[7] Darkins A, Ryan P, Kobb R, Foster L, Edmonson E, Wakefield B, Lancaster AE (2008). Care Coordination/Home Telehealth: the systematic implementation of health informatics, home telehealth, and disease management to support the care of veteran patients with chronic conditions. Telemed J E Health.

[8] Cahill S, Begley E, Faulkner JP, Hagen I (2007). TARA.

[9] Godwin KM, Mills WL, Anderson JA, Kunik ME (2013). Technology-driven interventions for caregivers of persons with dementia: a systematic review. Am J Alzheimers Dis Other Demen.

[10] Torkamani M, McDonald L, Saez AI, Kanios C, Katsanou M, Madeley L, Limousin PD, Lees AJ, Haritou M, Jahanshahi M, ALADDIN Collaborative Group (2014). A randomized controlled pilot study to evaluate a technology platform for the assisted living of people with dementia and their carers. J Alzheimers Dis.

[11] Evans J, Brown M, Coughlan T, Lawson G, Craven M (2015). A systematic review of dementia focused assistive technology.

[12] Mahoney DF, Coon DW, Lozano C (2016). Latino/Hispanic Alzheimer's caregivers experiencing dementia-related dressing issues: corroboration of the Preservation of Self model and reactions to a "smart dresser" computer-based dressing aid. Digit Health.

[13] Cahill S, Macijauskiene J, Nygård A, Faulkner J, Hagen I (2007). TARA.

[14] Orpwood R, Gibbs C, Adlam T, Faulkner R, Meegahawatte D (2005). The design of smart homes for people with dementia—user-interface aspects. Univ Access Inf Soc.

[15] Mirkovic J, Kristjansdottir OB, Stenberg U, Krogseth T, Stange KC, Ruland CM (2016). Patient insights into the design of technology to support a strengths-based approach to health care. JMIR Res Protoc.

[16] Ludden GD, van Rompay TJ, Kelders SM, van Gemert-Pijnen JE (2015). How to increase reach and adherence of web-based interventions: a design research viewpoint. J Med Internet Res.

[17] De Vito Dabbs A, Myers BA, Mc Curry KR, Dunbar-Jacob J, Hawkins RP, Begey A, Dew MA (2009). User-centered design and interactive health technologies for patients. Comput Inform Nurs.

[18] Pruitt J, Adlin T (2006). The Persona Lifecycle: Keeping People In Mind Throughout Product Design (Interactive Technologies).

[19] Johnson CM, Johnson TR, Zhang J (2005). A user-centered framework for redesigning health care interfaces. J Biomed Inform.

[20] Roth DL, Fredman L, Haley WE (2015). Informal caregiving and its impact on health: a reappraisal from population-based studies. Gerontologist.

[21] Astell AJ, Czarnuch S, Dove E (2018). System development guidelines from a review of motion-based technology for people with dementia or MCI. Front Psychiatry.

[22] Boman I, Persson A, Bartfai A (2016). First steps in designing an all-in-one ICT-based device for persons with cognitive impairment: evaluation of the first mock-up. BMC Geriatr.

[23] Matthews JT, Lingler JH, Campbell GB, Hunsaker AE, Hu L, Pires BR, Hebert M, Schulz R (2015). Usability of a wearable camera system for dementia family caregivers. J Healthc Eng.

[24] Dupuy B, Raffestin S, Matamouros S, Mani N, Popoff MR, Sonenshein AL (2006). Regulation of toxin and bacteriocin gene expression in Clostridium by interchangeable RNA polymerase sigma factors. Mol Microbiol.

[25] Levac D, Colquhoun H, O'Brien KK (2010). Scoping studies: advancing the methodology. Implement Sci.

[26] Rivera J, McPherson A, Hamilton J, Birken C, Coons M, Iyer S, Agarwal A, Lalloo C, Stinson J (2016). Mobile apps for weight management: a scoping review. JMIR Mhealth Uhealth.

[27] Braun V, Clarke V (2006). Using thematic analysis in psychology. Qual Res Psychol.

[28] Yang YT, Kels CG (2017). Ethical considerations in electronic monitoring of the cognitively impaired. J Am Board Fam Med.

[29] Chiu TM, Eysenbach G (2010). Stages of use: consideration, initiation, utilization, and outcomes of an internet-mediated intervention. BMC Med Inform Decis Mak.

[30] Wang RH, Sudhama A, Begum M, Huq R, Mihailidis A (2017). Robots to assist daily activities: views of older adults with Alzheimer's disease and their caregivers. Int Psychogeriatr.

[31] Lundberg S (2014). The results from a two-year case study of an information and communication technology support system for family caregivers. Disabil Rehabil Assist Technol.

[32] Mitseva A, Peterson CB, Karamberi C, Oikonomou LC, Ballis AV, Giannakakos C, Dafoulas GE (2012). Gerontechnology: providing a helping hand when caring for cognitively impaired older adults-intermediate results from a controlled study on the satisfaction and acceptance of informal caregivers. Curr Gerontol Geriatr Res.

[33] Martin S, Augusto JC, McCullagh P, Carswell W, Zheng H, Wang H, Wallace J, Mulvenna M (2013). Participatory research to design a novel telehealth system to support the night-time needs of people with dementia: NOCTURNAL. Int J Environ Res Public Health.

[34] Alm N, Astell A, Ellis M, Dye R, Gowans G, Campbell J (2004). A cognitive prosthesis and communication support for people with dementia. Neuropsychol Rehabil.

[35] Hamada T, Kuwahara N, Morimoto K, Yasuda K, Akira U, Abe S (2009). Preliminary study on remote assistance for people with dementia at home by using multi-media contents.

[36] Gibson G, Dickinson C, Brittain K, Robinson L (2015). The everyday use of assistive technology by people with dementia and their family carers: a qualitative study. BMC Geriatr.

[37] Price C (2007). Evaluation of an activity monitoring system for people with dementia. J Assist Technol.

[38] Olsson A, Engström M, Skovdahl K, Lampic C (2012). My, your and our needs for safety and security: relatives' reflections on using information and communication technology in dementia care. Scand J Caring Sci.

[39] Riikonen M, Paavilainen E, Salo H (2013). Factors supporting the use of technology in daily life of home-living people with dementia. Tech Disabili.

[40] Patterson T, McClean S, Langdon P, Zhang S, Nugent C, Cleland I (2014). A knowledge-driven approach to predicting technology adoption among persons with dementia. Conf Proc IEEE Eng Med Biol Soc.

[41] Brunete González A, Selmes M, Selmes J (2017). Can smart homes extend people with Alzheimer’s disease stay at home?. J Enabl Technol.

[42] Wan J, Byrne CA, OGrady MJ, OHare GM (2015). Managing wandering risk in people with dementia. IEEE Trans Human-Mach Syst.

[43] van den Heuvel E, Jowitt F, McIntyre A (2012). Awareness, requirements and barriers to use of Assistive Technology designed to enable independence of people suffering from Dementia (ATD). Tech Disabil.

[44] Cristancho-Lacroix V, Moulin F, Wrobel J, Batrancourt B, Plichart M, de Rotrou J, Cantegreil-Kallen I, Rigaud A (2014). A web-based program for informal caregivers of persons with Alzheimer's disease: an iterative user-centered design. JMIR Res Protoc.

[45] Nugent C, O’Neill S, Donnelly M, Parente G, Beattie M, McClean S, Scotney B, Mason S, Craig D (2011). Evaluation of video reminding technology for persons with dementia. Proceedings of the 9th international conference on Toward useful services for elderly and people with disabilities: smart homes and health telematics.

[46] Schroeter C, Mueller S, Volkhardt M, Einhorn E, Huijnen C, van den Heuvel H, van Berlo A, Bley A, Gross H (2013). Realization and user evaluation of a companion robot for people with mild cognitive impairments.

[47] O’Neill SA, Mason S, Parente G, Donnelly MP, Nugent CD, McClean S, Scotney B, Craig D (2010). Video reminders as cognitive prosthetics for people with dementia. Ageing Int.

[48] Boyd A, Synnott J, Nugent C, Elliott D, Kelly J (2017). Community-based trials of mobile solutions for the detection and management of cognitive decline. Healthc Technol Lett.

[49] Robinson L, Brittain K, Lindsay S, Jackson D, Olivier P (2009). Keeping In Touch Everyday (KITE) project: developing assistive technologies with people with dementia and their carers to promote independence. Int Psychogeriatr.

[50] Chou H, Yan S, Lin I, Tsai M, Chen C, Woung L (2012). A pilot study of the telecare medical support system as an intervention in dementia care: the views and experiences of primary caregivers. J Nurs Res.

[51] Williams K, Pennathur P, Bossen A, Gloeckner A (2016). Adapting telemonitoring technology use for older adults: a pilot study. Res Gerontol Nurs.

[52] Serafini JD, Damianakis T, Marziali E (2007). Clinical practice standards and ethical issues applied to a virtual group intervention for spousal caregivers of people with Alzheimer's. Soc Work Health Care.

[53] Evans N, Harris N, Kuppuswamy A (2011). A smarter future: technology to enhance an independent lifestyle for our future selves. Int J Ther Rehabil.

[54] Mahoney EL, Mahoney DF (2010). Acceptance of wearable technology by people with Alzheimer's disease: issues and accommodations. Am J Alzheimers Dis Other Demen.

[55] White EB, Montgomery P (2014). Electronic tracking for people with dementia: an exploratory study of the ethical issues experienced by carers in making decisions about usage. Dementia (London).

[56] Faucounau V, Riguet M, Orvoen G, Lacombe A, Rialle V, Extra J, Rigaud A (2009). Electronic tracking system and wandering in Alzheimer's disease: a case study. Ann Phys Rehabil Med.

[57] Faucounau V, Wu Y, Boulay M, Maestrutti M, Rigaud A (2009). Caregivers' requirements for in-home robotic agent for supporting community-living elderly subjects with cognitive impairment. Technol Health Care.

[58] Nestorov N, Stone E, Lehane P, Eibrand R (2014). Aspects of socially assistive robots design for dementia care.

[59] Bossen AL, Kim H, Williams KN, Steinhoff AE, Strieker M (2015). Emerging roles for telemedicine and smart technologies in dementia care. Smart Homecare Technol Telehealth.

[60] Sugihara T, Fujinami T, Miura M (2012). Approaches to incorporating assistive technologies into dementia care.

[61] Lopes P, Pino M, Carletti G, Hamidi S, Legué S, Kerhervé H, Benveniste S, Andéol G, Bonsom P, Reingewirtz S, Rigaud A (2016). Co-conception process of an innovative assistive device to track and find misplaced everyday objects for older adults with cognitive impairment: the TROUVE project. IRBM.

[62] Cavallo F, Aquilano M, Arvati M (2015). An ambient assisted living approach in designing domiciliary services combined with innovative technologies for patients with Alzheimer's disease: a case study. Am J Alzheimers Dis Other Demen.

[63] Kort H, van Hoof J (2014). Design of a website for home modifications for older persons with dementia. Tech Disabil.

[64] Verwey R, van Berlo M, Duymelinck S, Willard S, van Rossum E (2016). Development of an online platform to support the network of caregivers of people with dementia. Stud Health Technol Inform.

[65] Hattink B, Droes R, Sikkes S, Oostra E, Lemstra AW (2016). Evaluation of the Digital Alzheimer Center: testing usability and usefulness of an online portal for patients with dementia and their carers. JMIR Res Protoc.

[66] Godwin B (2012). The ethical evaluation of assistive technology for practitioners: a checklist arising from a participatory study with people with dementia, family and professionals. J Assist Technol.

[67] Boyd K, Nugent C, Donnelly M, Bond R, Sterritt R, Hartin P (2014). An investigation into the usability of the STAR training and re-skilling website for carers of persons with dementia. Conf Proc IEEE Eng Med Biol Soc.

[68] Pino M, Granata C, Legouverneur G, Rigaud A (2012). Assessing design features of a graphical user interface for a social assistive robot for older adults with cognitive impairment. Gerontechnology.

[69] Meiland FJ, Hattink BJ, Overmars-Marx T, de Boer ME, Jedlitschka A, Ebben PW, Stalpers-Croeze II, Flick S, van der Leeuw J, Karkowski IP, Dröes RM (2014). Participation of end users in the design of assistive technology for people with mild to severe cognitive problems; the European Rosetta project. Int Psychogeriatr.

[70] Egan K, Pot A (2016). Encouraging innovation for assistive health technologies in dementia: barriers, enablers and next steps to be taken. J Am Med Dir Assoc.

[71] Dröes R, Bentvelzen S, Meiland F, Craig D (2010). dementia-related and other factors to be taken into account when developing ICT support for people with dementia – lessons from field trials. Supporting People with Dementia Using Pervasive Health Technologies.

[72] Span M, Smits C, Jukema J, Groen-van de Ven L, Janssen R, Vernooij-Dassen M, Eefsting J, Hettinga M (2015). An interactive web tool to facilitate shared decision making in dementia: design issues perceived by caregivers and patients. Front Aging Neurosci.

[73] Boger J, Quraishi M, Turcotte N, Dunal L (2014). The identification of assistive technologies being used to support the daily occupations of community-dwelling older adults with dementia: a cross-sectional pilot study. Disabil Rehabil Assist Technol.

[74] Pino M, Benveniste S, Rigaud A, Jouen F (2013). Key factors for a framework supporting the design, provision, and assessment of assistive technology for dementia care. Assistive technology Research Series.

[75] O'Connor S, Bouamrane M, O'Donnell CA, Mair FS (2016). Barriers to co-designing mobile technology with persons with dementia and their carers. Stud Health Technol Inform.

[76] Archer N, Keshavjee K, Demers C, Lee R (2014). Online self-management interventions for chronically ill patients: cognitive impairment and technology issues. Int J Med Inform.

[77] Hellman R (2014). Assistive technologies for coping at home and increased quality of life for persons with dementia. eChallenges e-2014 Conference Proceedings.

[78] Becker S, Webbe F (2006). Use of handheld technology by older adult caregivers as part of a virtual support network.

[79] Glueckauf RL, Loomis JS (2003). Alzheimer's Caregiver Support Online: lessons learned, initial findings and future directions. NeuroRehabilitation.

[80] Pot A, Willemse B, Horjus S (2012). A pilot study on the use of tracking technology: feasibility, acceptability, and benefits for people in early stages of dementia and their informal caregivers. Aging Ment Health.

[81] Cristancho-Lacroix V, Wrobel J, Cantegreil-Kallen I, Dub T, Rouquette A, Rigaud A (2015). A web-based psychoeducational program for informal caregivers of patients with Alzheimer's disease: a pilot randomized controlled trial. J Med Internet Res.

[82] Kawamura T, Kono Y, Kidode M (2006). Towards a Wearable Cognitive Prosthesis to Support “What” and “Who” Type Memory Activities. http://www.irc.atr.jp/cpac2006/cpac_proceedings.pdf.

[83] Pot AM, Blom MM, Willemse BM (2015). Acceptability of a guided self-help Internet intervention for family caregivers: mastery over dementia. Int Psychogeriatr.

[84] Marziali E, Garcia LJ (2011). Dementia caregivers' responses to 2 Internet-based intervention programs. Am J Alzheimers Dis Other Demen.

[85] Epstein I, Aligato A, Krimmel T, Mihailidis A (2016). Older adults' and caregivers' perspectives on in-home monitoring technology. J Gerontol Nurs.

[86] Sarne-Fleischmann V, Tractinsky N, Dwolatzky T, Rief I (2011). Personalized reminiscence therapy for patients with Alzheimer's disease using a computerized system. Proceedings of the 4th International Conference on PErvasive Technologies Related to Assistive Environments.

[87] Boots LM, de Vugt ME, Withagen HE, Kempen GI, Verhey FR (2016). Development and Initial Evaluation of the Web-Based Self-Management Program "Partner in Balance" for Family Caregivers of People With Early Stage Dementia: An Exploratory Mixed-Methods Study. JMIR Res Protoc.

[88] Amiribesheli M, Bouchachia A (2015). Smart homes design for people with dementia.

[89] Begum M, Wang R, Huq R, Mihailidis A (2013). Performance of daily activities by older adults with dementia: the role of an assistive robot. IEEE Int Conf Rehabil Robot.

[90] Cooper C, Penders J, Procter PM (2016). Dementia and robotics: people with advancing dementia and their carers driving an exploration into an engineering solution to maintaining safe exercise regimes. Stud Health Technol Inform.

[91] Landau R, Auslander GK, Werner S, Shoval N, Heinik J (2011). Who should make the decision on the use of GPS for people with dementia?. Aging Ment Health.

[92] Navarro R, Favela J (2011). Usability assessment of a pervasive system to assist caregivers in dealing with repetitive behaviors of patients with dementia. Proceedings of the 4th International Conference on PErvasive Technologies Related to Assistive Environments.

[93] Ng J, Kong H (2016). Not All Who Wander Are Lost. Proceedings of the CHI Conference Extended Abstracts on Human Factors in Computing Systems - CHI EA '16.

[94] Hwang AS, Truong KN, Cameron JI, Lindqvist E, Nygård L, Mihailidis A (2015). Co-designing ambient assisted living (AAL) environments: unravelling the situated context of informal dementia care. Biomed Res Int.

[95] Holbø K, Bøthun S, Dahl Y (2013). Safe walking technology for people with dementia: what do they want?. Proceedings of the 15th International ACM SIGACCESS Conference on Computers and Accessibility.

[96] Kreuter MW, Wray RJ (2003). Tailored and targeted health communication: strategies for enhancing information relevance. Am J Health Behav.

[97] Rimer BK, Kreuter MW (2006). Advancing tailored health communication: a persuasion and message effects perspective. Journal of Communication.

[98] Noar SM, Benac CN, Harris MS (2007). Does tailoring matter? Meta-analytic review of tailored print health behavior change interventions. Psychol Bull.

[99] Czarnuch S, Mihailidis A (2011). The design of intelligent in-home assistive technologies: assessing the needs of older adults with dementia and their caregivers. Gerontechnology.

[100] Brankaert R, Snaphaan L, den Ouden E (2014). Stay in touch: an in context evaluation of a smartphone interface designed for people with dementia. International Workshop on Ambient Assisted Living 2014.

[101] Amiribesheli M, Bouchachia A (2016). Towards dementia-friendly smart homes.

[102] Alrige M, Chatterjee S (2015). Toward a Taxonomy of Wearable Technologies in Healthcare. Proceedings of the 10th International Conference on New Horizons in Design Science: Broadening the Research Agenda.

[103] Dixon BE, Zafar A, McGowan JJ (2007). Development of a taxonomy for health information technology. Stud Health Technol Inform.

[104] Boessen ABCG, Verwey R, Duymelinck S, van Rossum E (2017). An online platform to support the network of caregivers of people with dementia. J Aging Res.

[105] Beattie M, Hallberg J, Nugent C, Synnes K, Cleland I, Lee S (2014). A collaborative patient-carer interface for generating home based rules for self-management.

[106] Quintana E, Favela J (2012). Augmented reality annotations to assist persons with Alzheimers and their caregivers. Pers Ubiquit Comput.

[107] Altendorf A, Schreiber J (2015). Assistive technology in dementia care: methodological issues in research design. J Assist Technol.

[108] Giunti G (2018). 3MD for chronic conditions, a model for motivational mhealth design: embedded case study. JMIR Serious Games.

[109] AlMarshedi A, Wills GB, Ranchhod A (2015). he Wheel of Sukr: a framework for gamifying diabetes self-management in Saudi Arabia. Procedia Computer Science.

[110] Charles D, McDonough S (2014). A participatory design framework for the gamification of rehabilitation systems.

[111] Boletsis C, McCallum S, Landmark B (2015). The use of smartwatches for health monitoring in home-based dementia care.

[112] Newell AF (2011). Design and the Digital Divide: Insights from 40 Years in Computer Support for Older and Disabled People.

[113] Armstrong N, Nugent C, Moore G, Finlay D (2010). Developing smartphone applications for people with Alzheimer's disease. Proceedings of the 10th IEEE International Conference on Information Technology and Applications in Biomedicine.

[114] Austrom MG, Geros KN, Hemmerlein K, McGuire SM, Gao S, Brown SA, Callahan CM, Clark DO (2015). Use of a multiparty web based videoconference support group for family caregivers: innovative practice. Dementia (London).

[115] Carroll C, Chiodo C, Lin A, Nidever M, Prathipati J (2017). Robin: Enabling Independence For Individuals With Cognitive Disabilities Using Voice Assistive Technology. Proceedings of the 2017 CHI Conference Extended Abstracts on Human Factors in Computing Systems.

[116] Chiu TM, Eysenbach G (2011). Theorizing the health service usage behavior of family caregivers: a qualitative study of an internet-based intervention. Int J Med Inform.

[117] Ekström A, Ferm U, Samuelsson C (2017). Digital communication support and Alzheimer's disease. Dementia (London).

[118] Holthe T, Walderhaug S (2010). Older people with and without dementia participating in the development of an individual plan with digital calendar and message board. J Assist Technol.

[119] Hwang A, Truong K, Mihailidis A (2012). Using participatory design to determine the needs of informal caregivers for smart home user interfaces.

[120] Jordan P, Silva P, Nunes F, Oliveira R (2013). mobileWAY--A System to Reduce the Feeling of Temporary Lonesomeness of Persons with Dementia and to Foster Inter-caregiver Collaboration. Proceedings of the 2013 46th Hawaii International Conference on System Sciences.

[121] Kerssens C, Kumar R, Adams AE, Knott CC, Matalenas L, Sanford JA, Rogers WA (2015). Personalized technology to support older adults with and without cognitive impairment living at home. Am J Alzheimers Dis Other Demen.

[122] Kim H (2015). Understanding Internet use among dementia caregivers: results of secondary data analysis using the US caregiver survey data. Interact J Med Res.

[123] Landau R, Auslander GK, Werner S, Shoval N, Heinik J (2010). Families' and professional caregivers' views of using advanced technology to track people with dementia. Qual Health Res.

[124] Mahoney DF, Burleson W, Lozano C, Ravishankar V, Mahoney EL (2015). Prototype Development of a Responsive Emotive Sensing System (DRESS) to aid older persons with dementia to dress independently. Gerontechnology.

[125] Maier A, Özkil A, Bang M, Forchhammer B (2015). Remember to remember: A feasibility study adapting wearable technology to the needs of people aged 65 and older with Mild Cognitive Impairment (MCI) and Alzheimer's Dementia. Proceedings of International Conference on Engineering Design.

[126] Mann G, Oatley G (2017). Positive design of smart interactive fabric artifacts for people with dementia.

[127] Megges H, Freiesleben S, Jankowski N, Haas B, Peters O (2017). Technology for home dementia care: a prototype locating system put to the test. Alzheimers Dement (N Y).

[128] Mehrabian S, Extra J, Wu Y, Pino M, Traykov L, Rigaud A (2015). The perceptions of cognitively impaired patients and their caregivers of a home telecare system. Med Devices (Auckl).

[129] Mihailidis A, Blunsden S, Boger J, Richards B, Zutis K, Young L, Hoey J (2010). Towards the development of a technology for art therapy and dementia: definition of needs and design constraints. The Arts in Psychotherapy.

[130] Nauha L, Keränen NS, Kangas M, Jämsä T, Reponen J (2018). Assistive technologies at home for people with a memory disorder. Dementia (London).

[131] Pagán-Ortiz ME, Cortés DE, Rudloff N, Weitzman P, Levkoff S (2014). Use of an online community to provide support to caregivers of people with dementia. J Gerontol Soc Work.

[132] Peterson K, Hahn H, Lee AJ, Madison CA, Atri A (2016). In the Information Age, do dementia caregivers get the information they need? Semi-structured interviews to determine informal caregivers' education needs, barriers, and preferences. BMC Geriatr.

[133] Phua C, Roy P, Aloulou H, Biswas J, Tolstikov A, Foo V, Huang W, Feki M, Maniyeri J, Chu A, Xu D (2014). State-of-the-art assistive technology for people with dementia. InHandbook of Research on Ambient Intelligence and Smart Environments: Trends and Perspectives 2011 (pp. 300-319). IGI Global.

[134] Sawyer P, Sutcliffe A, Rayson P, Bull C (2015). Dementia and social sustainability: challenges for software engineering.

[135] Sugihara T, Fujinami T, Phaal R, Ikawa Y (2012). Gaps between assistive technologies and dementia care. 2012 Proceedings of PICMET '12: Technology Management for Emerging Technologies.

[136] Thorpe JR, Rønn-Andersen KV, Bień P, Özkil AG, Forchhammer BH, Maier AM (2016). Pervasive assistive technology for people with dementia: a UCD case. Healthc Technol Lett.

[137] Wolters MK, Kelly F, Kilgour J (2016). Designing a spoken dialogue interface to an intelligent cognitive assistant for people with dementia. Health Informatics J.

[138] Yamagata C, Coppola J, Kowtko M, Joyce S (2013). Mobile app development and usability research to help dementia and Alzheimer patients.

[139] Zhang S, McClean SI, Nugent CD, Donnelly MP, Galway L, Scotney BW, Cleland I (2014). A predictive model for assistive technology adoption for people with dementia. IEEE J Biomed Health Inform.

