# Supplementary material for: Factors Influencing the Adoption of Smart Health Technologies for People With Dementia and Their Informal Caregivers: Scoping Review and Design Framework
Source: JMIR Aging. 2019 Apr 30;2(1):e12192. doi: 10.2196/12192 (PMC6716546; doi:10.2196/12192)
Supplement: Multimedia Appendix 1 [file aging_v2i1e12192_app1.pdf]

“ANNEXES”

Annexes 1. Keywords search terms organisation groups table.

**Dementia related keywords**	**Caregiver related keywords**	**Smart Health technologies related keywords**
Dementia	*Caregiver*	*Connected health*
Alzheimer's disease	*Caregivers*	*mHealth*
Alzheimer disease	*Carer*	*eHealth*
Vascular dementia	*Carers*	*Medical informatics*
Cognitive decline		*Medical Informatics Applications*
Cognitive impairment		*Telecare/ Tele care*
Cognitive Dysfunction		*Telemedicine*
Senile		*Health technology*
		Biomedical Technology
		Health technologies
		e-care/ ecare
		Telehealth/ Tele health
		Online platform
		Online health
		Online system
		Online systems
		Mobile health
		Healthcare platform
		Health platform
		Sensor technology
		Sensor technologies
		Sensor monitoring
		Health monitoring
		Health monitor
		Health monitors
		Distance monitoring
		Distance health
		Monitoring technology
		Monitoring technologies
		Remote monitoring
		Remote monitor
		Remote monitors
		Remote care
		Virtual care
		Virtual health
		Telephone monitoring
		Assistive living
		Assisted living
		Assisted Living Facilities
		Assistive technology
		Assistive technologies
		Wearable
		Wearables
		e-medicine/ emedicine

		Digital health<br>*Digital monitoring*<br>*Digital monitor*<br>*Digital monitors*<br>*Digital literacy*<br>*Internet*

Annexes 2. Built search strategy.

[[[[[[[[[[[[[[[[[[dementia] OR "Dementia"[Mesh]] OR "alzheimer's disease"] OR "alzheimer disease"] OR "Alzheimer Disease"[Mesh]] OR "vascular dementia"] OR "Dementia, Vascular"[Mesh]] OR "cognitive decline"] OR "Cognitive Dysfunction"[Mesh]] OR senile] OR "Lewy bodies"] OR "Lewy Bodies"[Mesh]] OR "frontotemporal dementia"] OR "Frontotemporal Dementia"[Mesh]] OR "memory loss"] OR "Memory Disorders"[Mesh]] OR "cognitive impairment"]] AND [[[[[caregiver] OR "Caregivers"[Mesh]] OR caregivers] OR carer] OR carers]] AND [[["connected health"] OR mhealth] OR ehealth] OR "medical informatics"] OR "Medical Informatics Applications"[Mesh]] OR telecare] OR "tele care"] OR telemedicine] OR "Telemedicine"[Mesh]] OR "health technology"] OR "health technologies"] OR e-care] OR "e care"] OR telehealth] OR "tele health"] OR "medical devices"] OR "online platform"] OR "online health"] OR "online systems"] OR "online system"] OR "mobile health"] OR "Online Systems"[Mesh]] OR "mobile platform"] OR "healthcare platform"] OR "health platform"] OR "sensor technology"] OR "sensor technologies"] OR "sensor monitoring"] OR "health monitoring"] OR "health monitor"] OR "health monitors"] OR "distance monitoring"] OR "distance health"] OR "monitoring technology"] OR "monitoring technologies"] OR "remote monitoring"] OR "remote monitor"] OR "remote monitors"] OR "remote care"] OR "virtual care"] OR "virtual health"] OR "telephone monitoring"] OR "assistive living"] OR "assisted living"] OR "Assisted Living Facilities"[Mesh]] OR "assistive technology"] OR "assistive technologies"] OR wearable] OR wearables] OR "e-medicine"] OR "emedicine"] OR "digital health"] OR "digital monitoring"] OR "digital monitor"] OR "digital monitors"] OR "digital literacy"] OR internet] OR "Internet"[Mesh]] OR "health information technologies"] OR "health information technology"] OR "communication platform"] OR "health management platform"] OR "remote tracking"] OR "remote health monitoring"] OR "ambient monitoring"] OR "ambient monitor"] OR "ambient monitors"] OR "interactive health"] OR "Monitoring, Ambulatory"[Mesh]]

Annexes 3. Emerging themes results classification with reference to papers included in the review.

Main themes	Subthemes	Papers where are referenced
Theme 1: Attitudinal aspects.	Positive attitudes towards technology	Latino/Hispanic Alzheimer's caregivers experiencing dementia-related dressing issues: corroboration of the Preservation of Self model and reactions to a ''smart dresser'' computer-based dressing aid. 2016, Mahoney DF. et al.<br><br>The design of smart homes for people with dementia - User-interface aspects. 2005, Orpwood R. et al.<br><br>Stages of use: consideration, initiation, utilization, and outcomes of an internet-mediated intervention. 2010, Chiu ML. et al.<br><br>A cognitive prosthesis and communication support for people with dementia. 2004, Alm N. et al.<br><br>Digital communication support and Alzheimer's disease. 2017, Ekstrom A. et al.<br><br>Preliminary study on Remote Assistance for People with Dementia at Home by Using Multimedia Contents. 2009, Hamada T. et al.<br><br>An interactive web tool to facilitate shared decision making in dementia: Design issues perceived by caregivers and patients. 2014, Span M. et al.<br><br>Electronic tracking for people with dementia: An exploratory study of the ethical issues experienced by carers in making decisions about age. 2014, White EB. et al.<br><br>Ethical aspects of using GPS for tracking people with dementia: recommendations for practice. 2011, Landau R. et al.<br><br>Families' and Professional Caregivers' Views of Using Advanced Technology to Track People with Dementia. 2010, Landau R. et al.

		Ethical considerations in electronic monitoring of the cognitively impaired. 2017, Yang YT. et al.<br><br>“It gives me a sense of independence” - Findings from Ireland on the use of assistive technology for people with dementia. 2007, Cahill S. et al.<br><br>Robots to assist daily activities: views of older adults with Alzheimer’s disease and their caregivers. 2017, Wang RH. et al.<br><br>Electronic tracking system and wandering in Alzheimer's disease: a case study. 2009, Fauconau V. et al.<br><br>Caregivers' requirements for in-home robotic agent for supporting community-living elderly subjects with cognitive impairment. 2009, Fauconau V. et al.<br><br>Older people with and without dementia participating in the development of an individual plan with digital calendar and message board. 2010, Holthe T. et al.<br><br>Co-Conception Process of an Innovative Assistive Device to Track and Find Misplaced Everyday Objects for Older Adults with Cognitive Impairment: The TROUVE Project. 2016, Lopes P. et al.<br><br>The results from a two-year case study of an information and communication technology support system for family caregivers. 2014, Lundberg S. et al.<br><br>Dementia caregivers' responses to 2 Internet-based intervention programs. 2011, Marziali E. et al.<br><br>Gerontechnology: Providing a helping hand when caring for cognitively impaired older adults-intermediate results from a controlled study on the satisfaction and acceptance of informal caregivers. 2012, Mitseva A. et al.

		Development of an Online Platform to Support the Network of Caregivers of People with Dementia. 2016, Verwey R. et al.<br><br>Alzheimer's Disease rehabilitation using smartphones to improve patients' quality of life. 2013, Zmily A. et al.<br><br>Participatory research to design a novel telehealth system to support the night-time needs of people with dementia: NOCTURNAL. 2013, Martin S. et al.<br><br>The everyday use of assistive technology by people with dementia and their family carers: a qualitative study. 2015, Gibson G. et al.<br><br>Evaluation of an activity monitoring system for people with dementia. 2007, Price C. et al.<br><br>My, your and our needs for safety and security: relatives' reflections on using information and communication technology in dementia care. 2011, Olsson A. et al.<br><br>Can smart homes extend people with Alzheimer's disease stay at home? 2016, Brunete A. et al.<br><br>Managing wandering risk in people with dementia. 2015, Wan J. et al.<br><br>Factors supporting the use of technology in daily life of home-living people with dementia. 2013, Riikonen M. et al.<br><br>Awareness, requirements and barriers to use of Assistive Technology designed to enable independence of people suffering from Dementia [ATD]. 2012, Van den Heuvel E. et al.<br><br>A web-based program for informal caregivers of persons with Alzheimer's Disease: An iterative User-Centered Design. 2014, Cristancho-Lacroix V. et al.<br><br>Evaluation of video reminding technology for persons with dementia. 2011, Nugent C. et al.

		Realization and user evaluation of a companion robot for people with mild cognitive impairments. 2013, Schroeter C. et al.<br>Video Reminders as Cognitive Prosthetics for People with Dementia. 2011, O'Neill S. A. et al.<br><br>Community-based trials of mobile solutions for the detection and management of cognitive decline. 2017, Boyd A. et al.<br><br>Keeping In Touch Everyday [KITE] project: developing assistive technologies with people with dementia and their carers to promote independence. 2009, Robinson L. et al.<br><br>A pilot study of the telecare medical support system as an intervention in dementia care: the views and experiences of primary caregivers. 2012, Chou HK. et al.<br><br>Adapting Telemonitoring Technology Use for Older Adults: A Pilot Study. 2016, Williams K. et al.
	Negative attitudes towards technology	"It gives me a sense of independence" - Findings from Ireland on the use of assistive technology for people with dementia. 2007, Cahill S. et al.<br><br>Use of a multiparty web based videoconference support group for family caregivers: Innovative practice. 2015, Austrom M. G. et al.<br><br>A web-based program for informal caregivers of persons with Alzheimer's Disease: An iterative User-Centered Design. 2014, Cristancho-Lacroix V. et al.<br><br>Design and the Digital Divide. Insights from 40 years in Computer Support for Older and Disabled People. 2011, Alan F. Newel. et al.<br><br>An interactive web tool to facilitate shared decision making in dementia: design issues perceived by caregivers and patients. 2014, Span M. et al.

		The identification of assistive technologies being used to support the daily occupations of community-dwelling older adults with dementia: a cross-sectional pilot study. 2013, Boger J. et al.<br><br>Emerging roles for telemedicine and smart technologies in dementia care. 2015, Bossen AL. et al.<br><br>Caregivers' requirements for in-home robotic agent for supporting community-living elderly subjects with cognitive impairment. F2009, aucounau V. et al.<br><br>Electronic tracking for people with dementia: An exploratory study of the ethical issues experienced by carers in making decisions about usage. 2014, White EB. et al.<br><br>Electronic tracking system and wandering in Alzheimer's disease: A case study. 2009, Faucounau V. et al.<br><br>Ethical aspects of using GPS for tracking people with dementia: recommendations for practice. 2011, Landau R. et al.<br><br>A systematic review of dementia focused assistive technology. 2015, Evans J. et al.<br><br>Families' and Professional Caregivers' Views of Using Advanced Technology to Track People with Dementia. 2010, Landau R. et al.<br><br>The everyday use of assistive technology by people with dementia and their family carers: a qualitative study. 2015, Gibson G. et al.<br><br>Acceptance of wearable technology by people with Alzheimer's Disease: Issues and Accommodations. 2010, Mahoney EL. et al.<br><br>mobileWAY – A system to reduce the feeling of temporary lonesomeness of persons with dementia and to foster inter-caregiver collaboration. 2013, Jordan P. et al.<br><br>Not all who wander are lost: smart tracker for people with Dementia. 2016, Ng J. et al.

		Older adults' and caregivers' perspectives on in-home monitoring technology. 2016, Epstein I. et al.<br><br>Keeping In Touch Everyday [KITE] project: developing assistive technologies with people with dementia and their carers to promote independence. 2009, Robinson L. et al.<br><br>Technology in Dementia Care. 2007, Cahill S. et al.<br><br>The ethical evaluation of assistive technology for practitioners: a checklist arising from a participatory study with people with dementia, family and professionals. 2012, Godwin B. et al.<br><br>The use of smartwatches for health monitoring in home-based dementia care. 2015, Boletsis C. et al.<br><br>Ethical considerations in electronic monitoring of the cognitively impaired. 2017, Yang YT. et al.<br><br>Electronic tracking system and wandering in Alzheimer's disease: a case study. 2009, Fauconau V. et al.<br><br>Caregivers' requirements for in-home robotic agent for supporting community-living elderly subjects with cognitive impairment. 2009, Fauconau V. et al.<br><br>Understanding Internet Use Among Dementia Caregivers: Results of Secondary Data Analysis Using the US Caregiver Survey Data. 2015, Kim H. et al.<br><br>Technology for home dementia care: A prototype locating system put to the test. 2017, Megges H. et al.<br><br>Not all who wander are lost: Smart tracker for people with dementia. 2016, Ng J. et al.<br><br>Evaluation of video reminding technology for persons with dementia. 2011, Nugent C. et al.

		Video Reminders as Cognitive Prosthetics for People with Dementia. 2011, O'Neill S. A. et al.<br><br>The design of smart homes for people with dementia - User-interface aspects. 2005, Orpwood R. et al.<br><br>Realization and user evaluation of a companion robot for people with mild cognitive impairments. 2013, Schroeter C. et al.<br><br>Designing a spoken dialogue interface to an intelligent cognitive assistant for people with dementia. 2016, Wolters M.K. et al.<br><br>A Predictive Model for Assistive Technology Adoption for People With Dementia. 2014, Zhang S. et al.
Theme 2: Ethical issues.	Privacy concerns	A cognitive prosthesis and communication support for people with dementia. 2004, Alm N. et al.<br><br>A pilot study of the telecare medical support system as an intervention in dementia care: the views and experiences of primary caregivers. 2012, Chou HK. et al.<br><br>Emerging roles for telemedicine and smart technologies in dementia care. 2015, Bossen AL. et al.<br><br>Electronic tracking system and wandering in Alzheimer's disease: a case study. 2009, Fauconau V. et al.<br><br>Caregivers' requirements for in-home robotic agent for supporting community-living elderly subjects with cognitive impairment. 2009, Fauconau V. et al.<br><br>Participatory research to design a novel telehealth system to support the night-time needs of people with dementia: NOCTURNAL. 2013, Martin S. et al.

		Can smart homes extend people with Alzheimer's disease stay at home? 2016, Brunete A. et al.<br><br>Caregivers' requirements for in-home robotic agent for supporting community-living elderly subjects with cognitive impairment. 2009, Faucounau V. et al.<br><br>Electronic tracking for people with dementia: an exploratory study of the ethical issues experienced by carers in making decisions about usage. 2014, White EB. et al.<br><br>Evaluation of an activity monitoring system for people with dementia. 2007, Price C. et al.<br><br>Families' and Professional Caregivers' Views of Using Advanced Technology to Track People with Dementia. 2010, Landau R. et al.<br><br>The everyday use of assistive technology by people with dementia and their family carers: a qualitative study. 2015, Gibson G. et al.<br><br>Managing wandering risk in people with dementia. 2015, Wan J. et al.<br><br>The perceptions of cognitively impaired patients and their caregivers of a home telecare system. 2015, Mehrabian et al.<br><br>A pilot study on the use of tracking technology: Feasibility, acceptability, and benefits for people in early stages of dementia and their informal caregivers. 2012, Pot AM. et al.<br><br>Keeping In Touch Everyday [KITE] project: developing assistive technologies with people with dementia and their carers to promote independence. 2009, Robinson L. et al.<br><br>Clinical practice standards and ethical issues applied to a virtual group intervention for spousal caregivers of people with Alzheimer's. 2007, Serafini JD. et al.

		Dementia and Social Sustainability: Challenges for Software Engineering. 2015, Sawyer, P. et al.<br><br>The electronic, personalizable Rosetta system for dementia care: exploring the user-friendliness, usefulness and impact. 2016, Hattink B. et al.<br><br>Dementia and Social Sustainability: Challenges for Software Engineering. 2015, Sawyer P. et al.
	Autonomy concerns	"It gives me a sense of independence" - Findings from Ireland on the use of assistive technology for people with dementia. 2007, Cahill S. et al.<br><br>A web-based program for informal caregivers of persons with Alzheimer's Disease: An iterative User-Centered Design. 2014, Cristancho-Lacroix V. et al.<br><br>An interactive web tool to facilitate shared decision making in dementia: design issues perceived by caregivers and patients. 2014, Span M. et al.<br><br>The identification of assistive technologies being used to support the daily occupations of community-dwelling older adults with dementia: a cross-sectional pilot study. 2013, Boger J. et al.<br><br>Emerging roles for telemedicine and smart technologies in dementia care. 2015, Bossen AL. et al.<br><br>Caregivers' requirements for in-home robotic agent for supporting community-living elderly subjects with cognitive impairment. F2009, aucounau V. et al.<br><br>Electronic tracking for people with dementia: An exploratory study of the ethical issues experienced by carers in making decisions about usage. 2014, White EB. et al.<br><br>Electronic tracking system and wandering in Alzheimer's disease: A case study. 2009, Faucounau V. et al.

		Aspects of socially assistive robots design for dementia care. 2014, Nestorov N. et al.<br><br>Emerging roles for telemedicine and smart technologies in dementia care. 2015, Bossen AL. et al.<br><br>Ethical aspects of using GPS for tracking people with dementia: recommendations for practice. 2011, Landau R. et al.<br><br>A systematic review of dementia focused assistive technology. 2015, Evans J. et al.<br><br>Families' and Professional Caregivers' Views of Using Advanced Technology to Track People with Dementia. 2010, Landau R. et al.<br><br>The everyday use of assistive technology by people with dementia and their family carers: a qualitative study. 2015, Gibson G. et al.<br><br>Acceptance of wearable technology by people with Alzheimer's Disease: Issues and Accommodations. 2010, Mahoney EL. et al.<br><br>mobileWAY – A system to reduce the feeling of temporary lonesomeness of persons with dementia and to foster inter-caregiver collaboration. 2013, Jordan P. et al.<br><br>Not all who wander are lost: smart tracker for people with Dementia. 2016, Ng J. et al.<br><br>Older adults' and caregivers' perspectives on in-home monitoring technology. 2016, Epstein I. et al.<br><br>Keeping In Touch Everyday [KITE] project: developing assistive technologies with people with dementia and their carers to promote independence. 2009, Robinson L. et al.<br><br>Technology in Dementia Care. 2007, Cahill S. et al.<br><br>The ethical evaluation of assistive technology for practitioners: a checklist arising from a participatory study with people with dementia,

		family and professionals. 2012, Godwin B. et al.<br><br>Technology-driven interventions for caregivers of persons with dementia: a systematic review. 2013, Godwin B. et al.<br><br>The use of smartwatches for health monitoring in home-based dementia care. 2015, Boletsis C. et al.<br><br>Ethical considerations in electronic monitoring of the cognitively impaired. 2017, Yang YT. et al.<br><br>Development of an Online Platform to Support the Network of Caregivers of People with Dementia. 2016, Verwey R. et al.
	Data ownership concerns	Electronic tracking for people with dementia: An exploratory study of the ethical issues experienced by carers in making decisions about usage. 2014, White EB. et al.<br><br>Ethical considerations in electronic monitoring of the cognitively impaired. 2017, Yang YT. et al.<br><br>The electronic, personalizable Rosetta system for dementia care: exploring the user-friendliness, usefulness and impact. 2016, Hattink B. et al.
Theme 3: Technology-related challenges	Design	The identification of assistive technologies being used to support the daily occupations of community-dwelling older adults with dementia: a cross-sectional pilot study. 2013. Boger J. et al.<br><br>An Online Platform to Support the Network of Caregivers of People with Dementia. 2017, Boessen.<br><br>Smart Homes Design for People with Dementia. 2015, Amiribesheli M. et al.<br><br>A cognitive prosthesis and communication support for people with dementia. 2004, Alm N. et al.

		Towards the development of a technology for art therapy and dementia: Definition of needs and design constraints. 2010, Mihailidis A. et al.<br><br>Evaluation of the Digital Alzheimer Center: Testing Usability and Usefulness of an Online Portal for Patients with Dementia and Their Carers. 2016, Hattink B. et al.<br><br>The electronic, personalizable Rosetta system for dementia care: exploring the user-friendliness, usefulness and impact. 2016, Hattink B. et al.<br><br>Emerging roles for telemedicine and smart technologies in dementia care. 2015, Bossen AL. et al.<br><br>Remember to remember: A feasibility study adapting wearable technology to the needs of people aged 65 and older with mild cognitive impairment (MCI) and Alzheimer's dementia. 2015, Maier A. M. et al.<br><br>Co-Conception Process of an Innovative Assistive Device to Track and Find Misplaced Everyday Objects for Older Adults with Cognitive Impairment: The TROUVE Project. 2016, Lopes P. et al.<br><br>Developing smartphone applications for people with Alzheimer’s disease. 2010, Armstrong N. et al.<br><br>Can smart homes extend people with Alzheimer’s disease stay at home? 2016, Brunete A. et al.<br><br>Encouraging Innovation for Assistive Health Technologies in Dementia: Barriers, Enablers and Next Steps to Be Taken. 2016, Egan KJ. et al.<br><br>The everyday use of assistive technology by people with dementia and their family carers: a qualitative study. 2015, Gibson G. et al.<br><br>Latino/Hispanic Alzheimer’s caregivers experiencing dementia-related dressing issues:

		corroboration of the Preservation of Self model and reactions to a ‘‘smart dresser’’ computer-based dressing aid. 2016, Mahoney DF. et al.<br><br>Prototype Development of a Responsive Emotive Sensing System [DRESS] to aid older persons with dementia to dress independently. 2015, Mahoney DF. et al.<br><br>The perceptions of cognitively impaired patients and their caregivers of a home telecare system. 2015, Mehrabian S. et al.<br><br>My, your and our needs for safety and security: relatives’ reflections on using information and communication technology in dementia care. 2011, Olsson A. et al.<br><br>Design and the Digital Divide. Insights from 40 years in Computer Support for Older and Disabled People. 2011, Alan F. Newel. et al.<br><br>The design of smart homes for people with dementia - User-interface aspects. 2005, Orpwood R. et al.<br><br>Augmented reality annotations to assist persons with Alzheimers and their caregivers. 2013, Quintana E. et al.<br><br>Personalized reminiscence therapy for patients with Alzheimer's disease using a computerized system. 2011, Sarne-Fleischmann V. et al.<br><br>Community-based trials of mobile solutions for the detection and management of cognitive decline. 2017, Boyd A. et al.<br><br>Participation of end users in the design of assistive technology for people with mild to severe cognitive problems; the European Rosetta project. 2014, Meiland FJM. et al.<br><br>Dementia-Related and Other Factors to Be Taken into Account When Developing ICT Support for People with Dementia – Lessons from Field Trials. 2010, Dröes R-M. et al.

		An interactive web tool to facilitate shared decision making in dementia: design issues perceived by caregivers and patients. 2014, Span M. et al.<br><br>Online self-management interventions for chronically ill patients: cognitive impairment and technology issues. 2014, Archer N. et al.
	Digital Literacy	Awareness, requirements and barriers to use of Assistive Technology designed to enable independence of people suffering from Dementia [ATD]. 2012, Van den Heuvel E. et al.<br><br>My, your and our needs for safety and security: relatives' reflections on using information and communication technology in dementia care. 2011, Olsson A. et al.<br><br>The identification of assistive technologies being used to support the daily occupations of community-dwelling older adults with dementia: a cross-sectional pilot study. 2014, Boger J. et al.<br><br>A web-based program for informal caregivers of persons with Alzheimer's Disease: An iterative User-Centered Design. 2014, Cristancho-Lacroix V. et al.<br><br>A Knowledge-Driven Approach to Predicting Technology Adoption among Persons with Dementia. 2014, Patterson T. et al.<br><br>Assessing design features of a graphical user interface for a social assistive robot for older adults with cognitive impairment. 2012, Pino M et al.<br><br>Key Factors for a Framework Supporting the Design, Provision, and Assessment of Assistive Technology for Dementia Care. 2013, Pino M. et al.<br><br>Barriers to Co-Designing Mobile Technology with Persons with Dementia and Their Carers. 2016, O'Connor S. et al.

		An Ambient Assisted Living Approach in Designing Domiciliary Services Combined With Innovative Technologies for Patients With Alzheimer's Disease: A Case Study. 2015, Cavallo F. et al.<br><br>A systematic review of dementia focused assistive technology. 2015, Evans J. et al.<br><br>The everyday use of assistive technology by people with dementia and their family carers: a qualitative study. 2015, Gibson G. et al.<br><br>The design of intelligent in-home assistive technologies: Assessing the needs of older adults with dementia and their caregivers. 2011, Czarnuch S. et al.<br><br>Electronic tracking system and wandering in Alzheimer's disease: A case study. 2009, Faucounau V. et al.<br><br>Use of Handheld Technology by Older Adult Caregivers as Part of a Virtual Support Network. 2007, Becker SA. et al.<br><br>Adapting Telemonitoring Technology Use for Older Adults: A Pilot Study. 2016, Williams K. et al.<br><br>Keeping In Touch Everyday [KITE] project: developing assistive technologies with people with dementia and their carers to promote independence. 2009, Robinson L. et al.<br><br>In the Information Age, do dementia caregivers get the information they need? Semi-structured interviews to determine informal caregivers' education needs, barriers, and preferences. 2016, Peterson, K., et al.<br><br>The ethical evaluation of assistive technology for practitioners: a checklist arising from a participatory study with people with dementia, family and professionals. 2012, Godwin B. et al.
	Perceived usefulness	"It gives me a sense of independence" - Findings from Ireland on the use of assistive

		technology for people with dementia. 2007, Cahill S. et al<br><br>Emerging roles for telemedicine and smart technologies in dementia care. 2015, Bossen AL. et al.<br><br>The identification of assistive technologies being used to support the daily occupations of community-dwelling older adults with dementia: a cross-sectional pilot study. 2014, Boger J. et al.<br><br>Assistive technologies for coping at home and increased quality of life for persons with dementia. 2014, Hellman R. et al.<br><br>Usability of a Wearable Camera System for Dementia Family Caregivers. 2015, Matthews JT. et al.<br><br>Use of Handheld Technology by Older Adult Caregivers as Part of a Virtual Support Network. 2007, Becker SA. et al.<br><br>Alzheimer's Caregiver Support Online: Lessons learned, initial findings and future directions. 2003, Glueckauf RL.<br><br>First steps in designing an all-in-one ICT-based device for persons with cognitive impairment: evaluation of the first mock-up. 2016, Boman IL. et al.<br>Approaches to Incorporating Assistive Technologies into Dementia Care. 2012, Sugihara T. et al.<br><br>Design of a website for home modifications for older persons with dementia. 2014, Kort HSM. et al.<br>Development of an Online Platform to Support the Network of Caregivers of People with Dementia. 2016, Verwey R. et al.
Theme 4: Condition-related challenges	Cognitive decline	"It gives me a sense of independence" - Findings from Ireland on the use of assistive technology for people with dementia. 2007, Cahill S. et al

		A Knowledge-Driven Approach to Predicting Technology Adoption among Persons with Dementia. 2014, Patterson T. et al.<br><br>Dementia and Robotics: People with Advancing Dementia and Their Carers Driving an Exploration into an Engineering Solution to Maintaining Safe Exercise Regimes. 2016, Cooper C. et al.<br><br>Alzheimer's Caregiver Support Online: Lessons learned, initial findings and future directions. 2003, Glueckauf RL.<br><br>Acceptability of a guided self-help Internet intervention for family caregivers: mastery over dementia. 2015, Pot AM. et al.<br><br>First steps in designing an all-in-one ICT-based device for persons with cognitive impairment: evaluation of the first mock-up. 2016, Boman IL. et al.<br><br>Online self-management interventions for chronically ill patients: cognitive impairment and technology issues. 2014, Archer N. et al.<br><br>Dementia-Related and Other Factors to Be Taken into Account When Developing ICT Support for People with Dementia – Lessons from Field Trials. 2010, Dröes R-M. et al.<br><br>Awareness, requirements and barriers to use of Assistive Technology designed to enable independence of people suffering from Dementia [ATD]. 2012, Van den Heuvel E. et al.<br><br>Assessing design features of a graphical user interface for a social assistive robot for older adults with cognitive impairment. 2012, Pino M et al.<br><br>The identification of assistive technologies being used to support the daily occupations of community-dwelling older adults with dementia: a cross-sectional pilot study. 2013. Boger J. et al.

		Emerging roles for telemedicine and smart technologies in dementia care. 2015, Bossen AL. et al.<br><br>Community-based trials of mobile solutions for the detection and management of cognitive decline. 2017, Boyd A. et al.<br><br>An Investigation into the Usability of the STAR Training and Re-skilling Website for Carers of Persons with Dementia. 2014, Boys K. et al.<br><br>Stages of use: consideration, initiation, utilization, and outcomes of an internet-mediated intervention. 2010, Chiu ML. et al.<br><br>Theorizing the health service usage behavior of family caregivers: a qualitative study of an internet-based intervention. 2011, Chiu, T. M.<br><br>Electronic tracking for people with dementia: An exploratory study of the ethical issues experienced by carers in making decisions about usage. 2014, White EB. et al.<br><br>Acceptance of Wearable Technology by People With Alzheimer's Disease: Issues and Accommodations. 2010, Mahoney EL. et al.<br><br>Mobile App Development and Usability Research to Help Dementia and Alzheimer Patients. 2013, Yamagata C. et al.<br><br>Personalized Technology to Support Older Adults With and Without Cognitive Impairment Living at Home. 2015, Kerssens C. et al.<br><br>Towards Dementia-friendly Smart Homes. 2016, Amiribesheli M. et al.<br><br>Use of Handheld Technology by Older Adult Caregivers as Part of a Virtual Support Network. 2007, Becker SA. et al.<br><br>Encouraging Innovation for Assistive Health Technologies in Dementia: Barriers, Enablers and Next Steps to Be Taken. 2016, Egan KJ. et al.

		A pilot study of the telecare medical support system as an intervention in dementia care: the views and experiences of primary caregivers. 2012, Chou HK. et al.<br><br>Participation of end users in the design of assistive technology for people with mild to severe cognitive problems; the European Rosetta project. 2014, Meiland FJM. et al.<br><br>Factors supporting the use of technology in daily life of home-living people with dementia. 2013, Riikonen M. et al.<br><br>Stay in touch: an in context evaluation of a smartphone interface designed for people with dementia. 2014, Brankaert R. et al.<br><br>Assistive technologies at home for people with a memory disorder." Dementia (London). 2016, Nauha, L., et al.<br><br>A collaborative patient-carer interface for generating home based rules for self-management. 2015, Beattie, M., et al.<br><br>(State-of-the-art assistive technology for people with dementia. 2013, Phua, C., et al.<br><br>Ethical considerations in electronic monitoring of the cognitively impaired. 2017, Yang YT. et al.
	Aging and physical capabilities	“It gives me a sense of independence” - Findings from Ireland on the use of assistive technology for people with dementia. 2007, Cahill S. et al<br><br>A Knowledge-Driven Approach to Predicting Technology Adoption among Persons with Dementia. 2014, Patterson T. et al.<br><br>Alzheimer’s Caregiver Support Online: Lessons learned, initial findings and future directions. 2003, Glueckauf RL.<br><br>Acceptability of a guided self-help Internet intervention for family caregivers: mastery over dementia. 2015, Pot AM. et al.

		First steps in designing an all-in-one ICT-based device for persons with cognitive impairment: evaluation of the first mock-up. 2016, Boman IL. et al.<br><br>Online self-management interventions for chronically ill patients: cognitive impairment and technology issues. 2014, Archer N. et al.<br><br>Dementia-Related and Other Factors to Be Taken into Account When Developing ICT Support for People with Dementia – Lessons from Field Trials. 2010, Dröes R-M. et al.<br><br>Awareness, requirements and barriers to use of Assistive Technology designed to enable independence of people suffering from Dementia [ATD]. 2012, Van den Heuvel E. et al.<br><br>Assessing design features of a graphical user interface for a social assistive robot for older adults with cognitive impairment. 2012, Pino M et al.<br><br>The identification of assistive technologies being used to support the daily occupations of community-dwelling older adults with dementia: a cross-sectional pilot study. 2013. Boger J. et al.<br><br>Emerging roles for telemedicine and smart technologies in dementia care. 2015, Bossen AL. et al.<br><br>Community-based trials of mobile solutions for the detection and management of cognitive decline. 2017, Boyd A. et al.<br><br>An investigation into the usability of the STAR training and re-skilling website for persons with dementia. 2014, Boyd A. et al.<br><br>An Investigation into the Usability of the STAR Training and Re-skilling Website for Carers of Persons with Dementia. 2014, Boys K. et al.

		Stages of use: consideration, initiation, utilization, and outcomes of an internet-mediated intervention. 2010, Chiu ML. et al.<br><br>Theorizing the health service usage behavior of family caregivers: a qualitative study of an internet-based intervention. 2011, Chiu, T. M.<br><br>Electronic tracking for people with dementia: An exploratory study of the ethical issues experienced by carers in making decisions about usage. 2014, White EB. et al.<br><br>Acceptance of Wearable Technology by People With Alzheimer's Disease: Issues and Accommodations. 2010, Mahoney EL. et al.<br><br>Prototype Development of a Responsive Emotive Sensing System (DRESS) to aid older persons with dementia to dress independently. 2015, Mahoney D. F. et al.<br><br>Mobile App Development and Usability Research to Help Dementia and Alzheimer Patients. 2013, Yamagata C. et al.<br><br>Personalized Technology to Support Older Adults With and Without Cognitive Impairment Living at Home. 2015, Kerssens C. et al.<br><br>Towards Dementia-friendly Smart Homes. 2016, Amiribesheli M. et al.<br><br>Use of Handheld Technology by Older Adult Caregivers as Part of a Virtual Support Network. 2007, Becker SA. et al.<br><br>Encouraging Innovation for Assistive Health Technologies in Dementia: Barriers, Enablers and Next Steps to Be Taken. 2016, Egan KJ. et al.<br><br>A pilot study of the telecare medical support system as an intervention in dementia care: the views and experiences of primary caregivers. 2012, Chou HK. et al.<br><br>Participation of end users in the design of assistive technology for people with mild to

		severe cognitive problems; the European Rosetta project. 2014, Meiland FJM. et al.<br><br>Factors supporting the use of technology in daily life of home-living people with dementia. 2013, Riikonen M. et al.<br><br>Stay in touch: an in context evaluation of a smartphone interface designed for people with dementia. 2014, Brankaert R. et al.<br><br>Assistive technologies at home for people with a memory disorder." Dementia (London). 2016, Nauha, L., et al.<br><br>A collaborative patient-carer interface for generating home based rules for self-management. 2015, Beattie, M., et al.<br><br>(State-of-the-art assistive technology for people with dementia. 2013, Phua, C., et al.<br><br>Robin: Enabling independence for individuals with cognitive disabilities using voice assistive technology. 2017, Carroll C. et al.<br><br>Usability assessment of a pervasive system to assist caregivers in dealing with repetitive behaviors of patients with dementia. 2011, Navarro R. et al.<br><br>The design of smart homes for people with dementia - User-interface aspects. 2005, Orpwood R. et al.<br><br>Dementia and Social Sustainability: Challenges for Software Engineering. 2015, Sawyer P. et al.<br><br>Caregivers' requirements for in-home robotic agent for supporting community-living elderly subjects with cognitive impairment. 2009, Fauconau V. et al.

	Condition acceptance	"It gives me a sense of independence" - Findings from Ireland on the use of assistive technology for people with dementia. 2007, Cahill S. et al.<br><br>A web-based program for informal caregivers of persons with Alzheimer's Disease: An iterative User-Centered Design. 2014, Cristancho-Lacroix V. et al.<br><br>A web-based psychoeducational program for informal caregivers of patients with Alzheimer's disease: a pilot randomized controlled trial. 2015, Cristancho-Lacroix V. et al.<br><br>A pilot study on the use of tracking technology: Feasibility, acceptability, and benefits for people in early stages of dementia and their informal caregivers. 2012, Pot AM. et al.<br><br>Approaches to Incorporating Assistive Technologies into Dementia Care. 2012, Sugihara T. et al.<br><br>Awareness, requirements and barriers to use of Assistive Technology designed to enable independence of people suffering from Dementia [ATD]. 2012, Van den Heuvel E. et al.<br><br>The identification of assistive technologies being used to support the daily occupations of community-dwelling older adults with dementia: a cross-sectional pilot study. 2013, Boger J. et al.<br><br>Emerging roles for telemedicine and smart technologies in dementia care. 2015, Bossen AL. et al.<br><br>An Ambient Assisted Living Approach in Designing Domiciliary Services Combined With Innovative Technologies for Patients With Alzheimer's Disease: A Case Study. 2015, Cavallo F. et al.<br><br>Development and Initial Evaluation of the Web-Based Self-Management Program

		"Partner in Balance" for Family Caregivers of People With Early Stage Dementia: An Exploratory Mixed-Methods Study. 2016, Boots LMM. et al.<br><br>Development and Initial Evaluation of the Web-Based Self-Management Program "Partner in Balance" for Family Caregivers of People With Early Stage Dementia: An Exploratory Mixed-Methods Study. 2016, Boots LM. et al.<br><br>Electronic tracking system and wandering in Alzheimer's disease: A case study. 2009, Faucounau V. et al.<br><br>A systematic review of dementia focused assistive technology. 2015, Evans J. et al.<br><br>Families' and Professional Caregivers' Views of Using Advanced Technology to Track People with Dementia. 2010, Landau R. et al.<br><br>Gaps between Assistive Technologies and Dementia Care. 2012, Sugihara T. et al.<br><br>The everyday use of assistive technology by people with dementia and their family carers: a qualitative study. 2015, Gibson G. et al.<br><br>Latino/Hispanic Alzheimer's caregivers experiencing dementia-related dressing issues: corroboration of the Preservation of Self model and reactions to a 'smart dresser' computer-based dressing aid. 2016, Mahoney DF. et al.<br><br>Acceptance of Wearable Technology by People With Alzheimer's Disease: Issues and Accommodations. 2010, Mahoney EL. et al.<br><br>The perceptions of cognitively impaired patients and their caregivers of a home telecare system. 2015, Mehrabian et al.<br><br>Participation of end users in the design of assistive technology for people with mild to severe cognitive problems; the European Rosetta project. 2014, Meiland FJM. et al.

		Factors supporting the use of technology in daily life of home-living people with dementia. 2013, Riikonen M. et al.<br><br>Robots to assist daily activities: views of older adults with Alzheimer's disease and their caregivers. 2017, Wang RH. et al.<br><br>Stay in touch: an in-context evaluation of a smartphone interface designed for people with dementia. 2014, Brankaert R. et al.<br><br>Technology in Dementia Care. 2007, Cahill S. et al.<br><br>The design of intelligent in-home assistive technologies: Assessing the needs of older adults with dementia and their caregivers. 2011, Czarnuch S. et al.<br><br>Using participatory design to determine the needs of informal caregivers for smart home user interfaces. 2012, Hwang AS. et al.<br><br>Co-Designing Ambient Assisted Living (AAL) Environments: Unravelling the Situated Context of Informal Dementia Care. 2015, Hwang AS. et al.<br><br>Performance of Daily Activities by Older Adults with Dementia: The Role of an Assistive Robot. 2013, Begum M.<br><br>Pervasive assistive technology for people with dementia: a UCD case. 2016, Thorpe, J. R. et al.<br><br>Older adults' and caregivers' perspectives on in-home monitoring technology. 2016, Epstein I. et al.
Theme 5: Gaps	Market needs	The design of smart homes for people with dementia - User-interface aspects. 2005, Orpwood R. et al.<br><br>Towards a Wearable Cognitive Prosthesis to Support "What" and "Who" Type Memory Activities. 2006, Kawamura T. et a.

		A cognitive prosthesis and communication support for people with dementia. 2004, Alm N. et al.<br><br>Emerging roles for telemedicine and smart technologies in dementia care. 2015, Bossen AL. et al.<br><br>Managing wandering risk in people with dementia. 2015, Wan J. et al.
	Research	Gerontechnology: Providing a helping hand when caring for cognitively impaired older adults-intermediate results from a controlled study on the satisfaction and acceptance of informal caregivers. 2012, Mitseva A. et al.<br><br>An interactive web tool to facilitate shared decision making in dementia: Design issues perceived by caregivers and patients. 2014, Span M. et al.<br><br>A cognitive prosthesis and communication support for people with dementia. 2004, Alm N. et al.<br><br>An interactive web tool to facilitate shared decision making in dementia: design issues perceived by caregivers and patients. 2014, Span M. et al.<br><br>Emerging roles for telemedicine and smart technologies in dementia care. 2015, Bossen AL. et al.<br><br>Design and the Digital Divide. Insights from 40 years in Computer Support for Older and Disabled People. 2011, Alan F. Newel. et al.<br><br>Digital communication support and Alzheimer's disease. 2017, Ekstrom A. et al.<br><br>Design of a website for home modifications for older persons with dementia. 2014, Kort HSM. et al.<br><br>Participatory research to design a novel telehealth system to support the night-time

		needs of people with dementia: NOCTURNAL. 2013, Martin S. et al.<br><br>Positive design of smart interactive fabric artifacts for people with dementia. 2017, Mann G. et al.<br><br>Clinical practice standards and ethical issues applied to a virtual group intervention for spousal caregivers of people with Alzheimer's. 2007, Serafini JD. et al.<br><br>The ethical evaluation of assistive technology for practitioners: a checklist arising from a participatory study with people with dementia, family and professionals. 2012, Godwin B. et al.<br><br>Towards Dementia-friendly Smart Homes. 2016, Amiribesheli M. et al.<br><br>Smart Homes Design for People with Dementia. 2015, Amiribesheli M. et al.<br><br>Safe walking technology for people with dementia: What do they want? 2013, Holbø, K., et al.<br><br>Use of an online community to provide support to caregivers of people with dementia. 2014, Pagan-Ortiz, M. E., et al.<br><br>Towards a Wearable Cognitive Prosthesis to Support "What" and "Who" Type Memory Activities. 2006, Kawamura T. et a.
	Good practice guidelines	The results from a two-year case study of an information and communication technology support system for family caregivers. 2014, Lundberg S. et al.<br><br>Acceptability of a guided self-help Internet intervention for family caregivers: mastery over dementia. 2015, Pot AM. et al.<br><br>Dementia caregivers' responses to 2 Internet-based intervention programs. 2011, Marziali E. et al.<br><br>Aspects of socially assistive robots design for dementia care. 2014, Nestorov N. et al.

		Emerging roles for telemedicine and smart technologies in dementia care. 2015, Bossen AL. et al.<br><br>Ethical aspects of using GPS for tracking people with dementia: recommendations for practice. 2011, Landau R. et al.<br><br>A systematic review of dementia focused assistive technology. 2015, Evans J. et al.<br><br>The everyday use of assistive technology by people with dementia and their family carers: a qualitative study. 2015, Gibson G. et al.
